# Upper limb intention tremor assessment: opportunities and challenges in wearable technology

**DOI:** 10.1186/s12984-023-01302-9

**Published:** 2024-01-13

**Authors:** Natalia Paredes-Acuna, Daniel Utpadel-Fischler, Keqin Ding, Nitish V. Thakor, Gordon Cheng

**Affiliations:** 1 Institute for Cognitive Systems, Technical University of Munich, Arcisstraße 21, 80333 Munich, Germany; 2 Department of Neurology, School of Medicine, Technical University of Munich, Munich, Germany; 3 Department of Biomedical Engineering, Johns Hopkins School of Medicine, Baltimore, MD USA

**Keywords:** Tremor assessment, Intention tremor, Multiple sclerosis, Ataxia, Wearable sensors

## Abstract

**Background:**

Tremors are involuntary rhythmic movements commonly present in neurological diseases such as Parkinson's disease, essential tremor, and multiple sclerosis. Intention tremor is a subtype associated with lesions in the cerebellum and its connected pathways, and it is a common symptom in diseases associated with cerebellar pathology. While clinicians traditionally use tests to identify tremor type and severity, recent advancements in wearable technology have provided quantifiable ways to measure movement and tremor using motion capture systems, app-based tasks and tools, and physiology-based measurements. However, quantifying intention tremor remains challenging due to its changing nature.

**Methodology & Results:**

This review examines the current state of upper limb tremor assessment technology and discusses potential directions to further develop new and existing algorithms and sensors to better quantify tremor, specifically intention tremor. A comprehensive search using PubMed and Scopus was performed using keywords related to technologies for tremor assessment. Afterward, screened results were filtered for relevance and eligibility and further classified into technology type. A total of 243 publications were selected for this review and classified according to their type: body function level: movement-based, activity level: task and tool-based, and physiology-based. Furthermore, each publication's methods, purpose, and technology are summarized in the appendix table.

**Conclusions:**

Our survey suggests a need for more targeted tasks to evaluate intention tremors, including digitized tasks related to intentional movements, neurological and physiological measurements targeting the cerebellum and its pathways, and signal processing techniques that differentiate voluntary from involuntary movement in motion capture systems.

**Supplementary Information:**

The online version contains supplementary material available at 10.1186/s12984-023-01302-9.

## Background

### Introduction

Tremor is characterized as an involuntary, rhythmic, oscillatory movement of a body part [1], and it can manifest as a symptom of various neurological diseases, including essential tremor (ET), Parkinson’s disease (PD), and multiple sclerosis (MS). The categorization of tremors is based on clinical factors such as anatomical distribution, activation conditions, amplitude, frequency, and underlying etiology. Within the scope of this review, tremors will be classified according to their activation condition and corresponding neurological symptoms and diseases.

Tremor can be classified into two main categories: rest tremor [2], characterized by nonvoluntary activation that occurs when the individual is attempting to rest and is commonly observed in people with PD. In contrast, action tremor [1] involves voluntary movement. Action tremor can be further classified into two subtypes: postural tremor, which occurs when the subject maintains a position against gravity, and kinetic tremor, which is associated with any voluntary movement that can be constant (simple kinetic), specific to a particular activity, such as writing (task-specific), or that increases as the individual approaches a goal or visual target (intention tremor). Intention tremor refers to a rise in the amplitude of tremors when visually guided movements are made toward a target, especially when nearing it. This type of tremor can also be coupled with task-specific tremor as the individual performs targeted movements, for example, during drawing (Archimedes Spiral tests). Intention tremor is believed to be correlated with cerebellar pathology, its connected pathways, or both, and it is a common symptom in people with, for example, MS [3]. It is estimated that 25–60% of people with MS experience postural and intention tremor [4], which typically occurs in the upper limbs at a frequency of 3–4 Hz [3]. However, other types of tremors, such as rest, simple kinetic, and task-specific tremors, are not frequently observed in MS [5].

Assessing tremors in patients with neurological diseases is crucial for determining disease progression and the effectiveness of medical treatments. Traditionally, clinicians use various clinical tests to identify tremor type and severity in patients. However, with the advancement of wearable technologies, such as smartphones, smartwatches, and sophisticated muscle sensors, there are now quantifiable ways to measure movement and tremor. Although wearable technology is a promising approach for quantifying tremors, identifying relevant features for each type of tremor is necessary for practical use. Recent research has shown that analyzing tremor amplitude and frequency makes it possible to differentiate between different movement disorders such as ET and PD versus healthy controls, classify tremor severity, and correlate it with traditional qualitative-scored neurological tests [6]. However, the changing nature of intention tremors, whose amplitude depends on the movement intention of the patient, makes it difficult to quantify this type of tremor and extract valuable features using the current approaches.

Identifying and analyzing intention tremors can greatly aid disease progression monitoring and intervention efficacy assessment. This review examines the advancement of upper limb tremor assessment technology, methodology, and future directions for algorithm and sensor development to improve quantification of tremor in general and intention tremor specifically.

### Neurological tests for tremor assessment correlation and comparison

Researchers evaluate tremor assessment technologies by performing specific tasks that amplify the targeted tremor type. These tasks are based on tests used in clinical practice to assess upper limb impairments. Table 1 displays the most common clinical tests used to correlate or as a reference for evaluating assessment technologies. The Fahn-Tolosa-Marin Tremor Scale (FTMRS) [7] and the Essential Tremor Rating Assessment Scale (TETRAS) [8] are frequently used to quantify rest, postural, and kinetic tremor, including tremor during activities of daily living (ADLs). When the technology is tailored for a single population, e.g., people with PD, a more disease-specific test such as the Movement Disorder Society Unified Parkinson’s Disease Rating Scale, Part III Motor Examination (UPDRS-III) [9] is used for correlation purposes.

**Table 1:** Common neurological tests and tasks used in clinical practice to assess tremor

Name	Tremor type/function	Tasks								
		Rest	Posture against gravity	Write	Archimedes spiral	FT	FTN	Finger chase	ADLs	Other
FTMRS [7]	Rest, postural, kinetic	X	X	X			X		X	
TETRAS [8]	Postural, kinetic		X	X	X		X		X	X
UPDRS-III [9]	Rest, postural, kinetic	X	X			X	X			
SARA [10]	Postural, kinetic, ataxia		X				X	X		X

*FTMRS* Fahn-Tolosa-Marin Tremor Scale, *TETRAS* Essential Tremor Rating Assessment Scale, *UPDRS-III* Movement Disorder Society Unified Parkinson’s Disease Rating Scale, Part III Motor Examination, *SARA* Scale for the Assessment and Rating of Ataxia, *FT* Finger Tapping, *FTN* Finger To Nose test, *ADLs* Activities of Daily Living

Another example of a disease-specific test is the Scale for the Assessment and Rating of Ataxia (SARA) test [10], which focuses on cerebellar ataxia. SARA includes the finger to nose test (FTN) and the finger chase test, which specifically evaluates intention tremor.

**Fig. 1:**
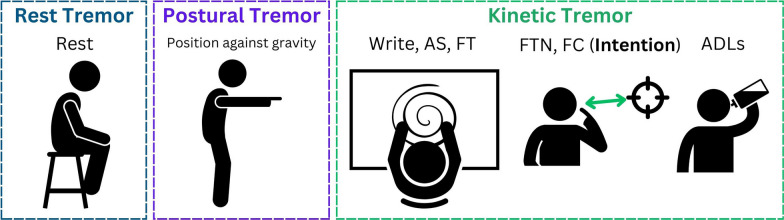
Rest tremors are evaluated using supported positions, postural tremors with no support, kinetic tremors through tasks such as writing, finger tapping (FT), and Activities of Daily Living (ADLs), and intention tremors using tasks such as finger to nose test (FTN) and finger chase (FC) tests

In summary, clinical tests include different tasks assessing tremor severity depending on their type (see Fig. 1):

- *Rest tremor*: Sitting with fully supported arms against gravity.
- *Postural tremor*: Maintaining a specific posture against gravity, for example, stretching arms to the front so that the subject maintains their elbows stretched against gravity; or shoulder abduction with elbows flexed and hands held in a pronated position resembling a 'wing-beating' posture.
- *Kinetic tremor*: Simple kinetic and task-specific tremors are evaluated using tasks such as handwriting, Archimedes spirals drawings, and finger tapping (FT), as well as ADLs involving whole-body movement, such as pouring drinks, eating, and dressing. Intention tremor severity can be measured using the finger to nose test (FTN). In this test, the subject touches their nose and then the examiner’s finger, with the tremor amplitude expected to increase as the hand approaches the finger. Intention tremor can also be assessed using the finger chase test, where the examiner performs sudden fast pointing movements in a frontal plane. At the same time, the subject follows with their finger as quickly and accurately as possible.

### Literature search and data extraction

**Fig. 2:**
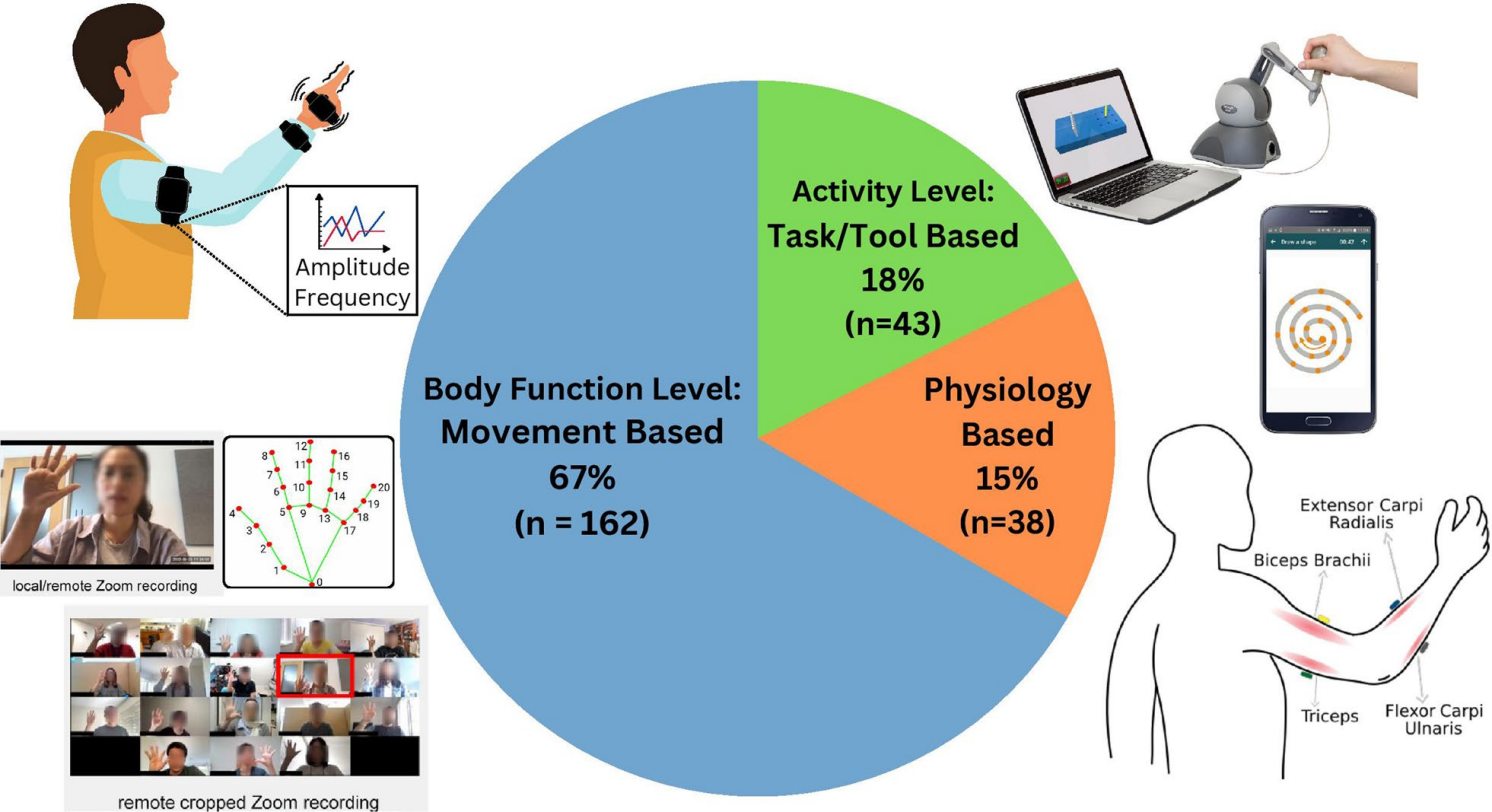
Types of tremor assessment technologies include activity level tasks and tools such as tablets and smartphones for drawing, physiological technologies such as surface electromyography (EMG) and electroencephalogram (EEG), and body function level movement-based technologies such as inertial measurement units (IMUs) and camera systems for measuring upper limb pose and movement. Figures adapted from [13, 14] used under CC BY 4.0 and from [15] used under granted copyright by CCC RightsLink

This review was primarily conducted using the Preferred Reporting Items for Systematic Reviews and Meta-Analyses (PRISMA) scoping review checklist (see Additional file 2). In this review, we were interested in finding studies examining quantifiable upper limb tremor assessment strategies accessible to clinicians and patients without highly specialized equipment. To determine the criteria for inclusion and exclusion, we conducted a comprehensive search on PubMed and Scopus with the following title/abstract terms ("tremor") AND ("assessment" OR "measurement" OR "evaluation" OR "detection" OR "quantification" OR "monitoring" OR "correlation" OR "estimation" or "discrimination" OR "analysis" OR "differentiation" OR "classification") AND ("technology" OR "sensor" OR "device" OR "quantification") (last search date: 10 July 2023) (see Additional file 5 for the detailed search strings). Further publications were identified from the list of references of relevant papers and relevant review papers found in our search [6, 11, 12]. After screening the articles for relevance and eligibility, we excluded studies that (1) did not focus on upper limb impairment, (2) focused on upper limb symptoms that explicitly excluded tremor, (3) only used clinical tests and clinician evaluation without any sensor or any automated tool, (4) the type of technology is not portable or usable outside of specialized rooms (e.g., functional magnetic resonance (fMRI) or magnetoencephalography (MEG)) or are invasive, (5) only evaluated healthy subjects, (6) interventional studies using damping tools, such as exoskeletons or functional electrical stimulation (FES), (7) preprints, prospective studies, and not peer-reviewed, and (8) not written in English. The remaining studies, 243 publications (see the details on data extraction in Additional file 3), were analyzed to identify common themes and establish criteria based on the type of sensors, number of subjects, technology, methodology, purpose, and year of publication. According to our screened papers, tremor assessment technologies can be classified into three distinct types, as depicted in Fig. 2 and classified in the table of Additional file 1 and the database found in Additional file 4:

i. *Activity level: Based on tools and digitized tasks*, using smartphones or tablets, assessment is made through manipulanda or touch-based games.
ii. *Based on physiological sensors,* physiological measurements are used to detect and differentiate tremors using surface electromyography (EMG) sensors, muscle activation following motor unit recruitment, and electroencephalogram (EEG) measuring the brain's electrical activity from the scalp.
iii. *Body function level: Movement based on motion capture systems*, the tremor and the posture of the subject’s upper limbs are captured using accelerometers, gyroscopes, inertial measurement units (IMUs), electromagnetic tracking, or camera systems, with or without markers.

## Technologies for tremor assessment

The following sections will discuss the different assessment technologies and algorithms to quantify tremors. The studies in this section have been classified in detail according to sensor type, patient population, and tremor type in Additional files 1 and 4. We encourage the readers to consider this chapter together with those additional files. Table 2 presents an overview of the tools discussed in this chapter and the main type of tremor assessed with them.

**Table 2:** Summary of type of technology and main targeted tremor discussed in Chapter 2

Section	Type	Main targeted tremor
2.1 Algorithms	Fourier transform (FFT), power spectral analysis (PSD), wavelet decomposition (DWT), and Hilbert-Huang transform (HHT)	All types
2.2 Tool based	Smart pens and manipulanda with embedded IMUs and force sensors	Task-specific tremor
2.3 Task based	Digitized drawings such as Archimedes Spirals, and different types of shapes. Smartphone games where the subject maintain objects in equilibrium	Task-specific and intention tremor
2.4 Physiology based	Muscle activity using EMG and MMG	Rest and postural tremor, discrimination between ET and PD
2.4 Physiology based	Brain activity using EEG	Rest and postural tremor
2.5 Movement based	Accelerometers, gyroscopes, magnetometers and IMUs (sensor fusion)	Rest, postural, and intention tremor
2.6 Movement based	Cameras	Rest, postural, and intention tremor

*EMG* electromyography, *MMG* mechanomyography, *PD* Parkinson’s disease, *ET* essential tremor, *IMUs* inertial measurement units

### Signal processing to quantify and analyze tremors

Tremor assessment technologies measure physical parameters and transform them into electronic signals. For instance, accelerometers placed on the subject’s hand analyze the frequency components of arm acceleration to detect tremors. Signal processing techniques are necessary to remove noise and measure various movement features. The publications in our review employ different algorithms and feature extraction methods based on signal processing techniques for tremor detection. To detect tremors, measurements are typically transformed from the time domain to the frequency domain, focusing on tremor frequencies (2–10 Hz) compared to regular movement. Fast Fourier transform (FFT) and power spectral distribution (PSD) analysis are commonly used. The FFT provides information about the amplitude and phase of individual frequency components in a signal, while the PSD offers insights into the power distribution across different frequency bands. The PSD is especially suitable for comparing signals of varying lengths because it focuses on the frequency distribution regardless of the signal length. In contrast, the FFT is dependent on the signal length.

In addition to the FFT and PSD, decomposing electronic signals in both time and frequency is advantageous, particularly for analyzing changes in frequency strength over time. The discrete wavelet transform (DWT) and Hilbert-Huang transform (HHT) [16] can be helpful for this. The DWT decomposes a signal into wavelets of different frequencies, scales, and orientations, making it more efficient to simultaneously analyze both frequency and time information, more robust to noise, and computationally efficient. On the other hand, the HHT decomposes a signal into its intrinsic mode functions (IMFs) using empirical mode decomposition (EMD) [17] and is better suited for analyzing nonstationary signals with precise time–frequency information. However, it may require more processing power. Thus, DWT and EMD are valuable tools to decompose voluntary and involuntary movement.

### Manipulanda and technical tools to quantify tremors

One approach for assessing tremors involves using tools with embedded sensors that can measure the direction, speed, and force of movement [18–24]. Researchers have utilized tools such as pens [25–30] with embedded IMUs and load cells to quantify tremor amplitude while users hold it, attach it to their hands, or write with it. An advantage of embedded sensor tools is their ability to identify different features in virtual tasks [31–33]. For example, the Virtual Peg Insertion Test (VPIT), based on the 9HPT [34] test, employs a manipulandum with force sensors in a virtual game environment and serves as a digital health metric for predicting the response to neurorehabilitation interventions in neurological disorders.

Kanzler et al. [13, 35] identified several features and studied their correlation to clinical tests. They found a high correlation between the SARA test and velocity and path length features in relation to intention tremor. Manipulanda have also been used to elicit intention tremor during goal-directed movements; for example, Feys et al. [36, 37] conducted studies involving people with MS (pwMS) and intention tremors, where they observed more significant target overshoot and unsteady eye fixation during goal-directed movement tasks.

Overall, pens with embedded IMUs have shown promise in measuring different types of tremors, particularly during task-specific movements such as writing or drawing [28]. However, wearable sensors may be more suitable and sensitive for measuring steady tremors than tools. On the other hand, analyzing digital features in addition to traditional completion time in tests such as the 9HPT could provide further insight into the characteristics of intention tremor. However, focused symptom testing is necessary to determine the effectiveness of these digital features in measuring intention tremor. Therefore, studies that specifically focus on it, using manipulanda in tasks similar to the finger chase test [36–38], would be advantageous; however, a quantification of intensity and its test correlation would still be required for future studies.

### From measuring the duration of completion to quantifying the drawn lines

Digitized drawing tests, such as writing or drawing shapes on tablets or smartphones, offer advantages over traditional methods of assessing tremors. These tests allow for the quantification of drawn lines in terms of time and extraction of different features. The assessment of digitized drawings often involves calculating the power spectral density (PSD) of the drawing position, velocity, or acceleration to determine the frequency ranges of the movement. This can help distinguish subjects with tremors, who are expected to have distinguishable spectra at higher frequencies (> 2 Hz), from those without tremors. Digitizing tablets have been used to assess tremor by analyzing writing and drawing shapes and AS [39–49], as well as combining it with FT [50–53]. Studies have shown that the frequency spectrum of velocity profiles in digitized Archimedes spirals drawings is a reliable measure of tremor intensity and more accurate than traditional visual rating methods [54].

Smartphone apps offer greater accessibility and flexibility for at-home testing compared to tablets since individuals are more likely to possess a smartphone than a tablet. Furthermore, the choice between smartphones and tablets can affect the reproducibility and intravariability of results, and more straightforward tests may be preferred for smartphone-based MS assessment [55]. This could be advantageous, especially in using small screens where drawings are limited due to space. These approaches include drawing simpler shapes than Archimedes spirals [14, 56–58], tilting a smartphone to maintain an objective in position using the smartphone accelerometers [59–61], and finger tapping (FT) to assess upper limb impairment [62, 63].

Regarding intention tremor, Erasmus et al. [64] pioneered this method for quantification of ataxic symptoms in MS. They tested it in a large cohort of 342 pwMS where they drew an’8’ shape in a tablet. Consequently, Feys et al. [65] investigated the validity and reliability of drawing regular and squared Archimedes spirals on a tablet as a test for tremor severity. They successfully differentiate pwMS with intention tremor from pwMS with no tremor and healthy subjects (HS) by comparing the radial and tangential velocity PSD in the 3–5 Hz frequencies with FTMRS scores. Archimedes spirals drawings have also proven to be a good measure to identify the presence of intention tremor in pwMS by comparing it with FTN, 9HPT, and BBT [66]. Measuring the segment rate, i.e., the number of times the pen changes from the upward to the downward direction, is the feature that correlates more to visually inspected intention tremor. The advantage of this metric is probably related to the fact that the segment rate increases as the frequency of the movement increases, suggesting that intention tremor could also be detected by analyzing the PSD of the Archimedes spirals movement, as proven by Creagh et al. [56] during the DaS test.

In summary, digitized drawings and app-based games are accessible tools to quantify tremors that could be used in clinics and at home. Tasks such as Archimedes spirals are very effective in eliciting tremors in various neurological diseases. However, it is still unclear how this task is related to intention tremor. Further analysis and correlation to intention tremor tasks, for example, using it in combination with the SARA test, would provide a deeper understanding of its relation to intentional movements.

### Physiological measurements: discriminating between different neurological diseases

Surface electromyography (EMG), measuring muscle electrical activity, and mechanomyography (MMG), measuring surface oscillations produced by motor units, are used to analyze muscle activation patterns in upper limb tremors. In the 80–90s, EMG was used to detect tremors using FFT and PSD in subjects with neurological disorders [67–69]. EMG has been used to distinguish muscle activation depending on the neurological disease [70–72]; for example, Nisticò and Vescio et al. [73, 74] showed that during rest tremor, the activation of antagonist muscles is synchronous in subjects with ET and alternating in those with PD. EMG and accelerometer/IMU combinations [75–83] have been extensively used to discriminate PD, ET [84–89], physiological tremor (PH) [90, 91], psychogenic tremor [92, 93], advanced ET [94], and MS [95] from each other by using ML techniques on DWT and HT signal decomposition during, in its majority, stretch and steady positions. MMG [96] was recently used with EMG, force sensors, and IMUs to detect tremor differences in PD after deep brain stimulation [97].

Electroencephalogram (EEG) measures the brain's electrical activity from the scalp, providing excellent temporal resolution. However, its low spatial resolution poses a challenge in precisely identifying activity in different brain structures. Despite this drawback, EEG is a valuable tool for evaluating motor tasks [98], as long as the influence of movement artifacts is carefully considered. EEG has been used to explore the involvement of the cerebellum in conditions such as spinocerebellar and cerebellar AT [99, 100], as well as ET in comparison with PD [101, 102], HS [103], and people with age-related tremors (ART) [104]. These studies consistently demonstrate a strong involvement and oscillations of cerebellar activity in ET and PD. Excessive oscillations in cerebellar EEG have been correlated with tremor intensity in ET [105, 106], while increased oscillations in the theta band of cerebellar EEG have been observed in PD [107]. EEG has also been employed to assess the effects of transcranial magnetic stimulation (TMS) therapy in individuals with multiple system atrophy cerebellar subtypes (MSA-C) [108], showing higher cerebello-frontal connectivity and a negative correlation to SARA.

EMG and MMG measurements have effectively been used to differentiate tremor pattern activations in different neurological conditions, even when the subjects perform the same type of activity. These results suggest that muscular activity could be a powerful tool to understand how tremor is propagated and where it is localized. On the other hand, the mentioned studies have emphasized the importance of EEG in studying the involvement of the cerebellum in movement disorders, which could provide valuable insights into the underlying pathophysiology of intention tremor and potential treatment strategies.

### Inertial-based recordings using acceleration, orientation, and sensor fusion algorithms

Inertial measurement units (IMUs), consisting of accelerometers, gyroscopes, and magnetometers, measure linear acceleration, angular velocity, and magnetic field strength, respectively. As these signals vary depending on the orientation of the sensor, IMUs have become increasingly prevalent in modern technology applications. These sensors can be positioned on different parts of the limbs, such as the wrist, hand, or fingers, to analyze movement by measuring the acceleration, velocity, and orientation of the limbs. Furthermore, suppose multiple IMUs are used on each limb segment, i.e., hand, forearm, upper arm, and trunk. In that case, it is possible to extract the limb's position relative to the trunk and measure additional features such as range of motion and movement synergy.

In the past, accelerometers, gyroscopes, and magnetometers were available as separate components, and smartphones typically only included accelerometers due to cost considerations. At the end of the last century, accelerometers were used to detect tremors [109–113], quantify medication efficacy [114] in PD, and analyze intention tremors in patients with cerebellar pathology [115]. Accelerometers attached to the hands or wrist either in single form [116–144] or in the form of a smartwatch [145–156] or smartphone [157–165] have been extensively used to quantify tremors in different neurological diseases [166, 167], either by analyzing acceleration frequency [88, 168] and amplitude [169] or by using machine learning methods to classify measurements according to tremor type [170–173]. Gyroscopes can detect changes in angular velocity and measure the angular movement of a body part. Analogous to accelerometers, gyroscopes have also been used individually [174–178], in smartphones [179] and smartwatches [180–182] to decompose tremorous and voluntary movement using different signal processing techniques such as EMD, HHT [183, 184], WFLC, and EKF [185]. Other types of motion detection sensors, such as force transducers [186–188] or electromagnetic sensors [189–194], have been proposed to track tremors in ET, PD, and MS.

The miniaturization of IMUs has enabled the direct measurement of tremors on distal limbs using a single chip. Although some studies have utilized both accelerometers and gyroscopes [96, 97, 195–230] to gain insight into tremorous movements, only a portion of them have employed sensor fusion algorithms to integrate these data and improve measurement reliability [131, 231–255]. Sensor fusion filters are used in IMUs to combine data from multiple sensors and improve the accuracy and reliability of the measurements. Their output is no longer angular velocity or acceleration but the IMU orientation relative to a predefined reference. Popular filters include the Madgwick filter and extended Kalman filter (EKF). The Madgwick filter is computationally efficient, using quaternions to combine accelerometer, gyroscope, and magnetometer data for orientation estimation. In contrast, the EKF employs a mathematical model and Bayesian inference to estimate the system state by fusing data from multiple sensors.

Overall, measuring acceleration and angular velocity, using electromagnetic tracking to track upper limb movement, or using a combination of sensors embedded in IMUS has proven to be a popular and straightforward method for measuring tremors. To achieve a more accurate and comprehensive understanding of tremorous movement, future research should use sensor fusion algorithms, which are currently underutilized (less than 39% of the studies using IMUs). This approach would enable researchers to calculate limb position, velocity, and acceleration without the noise drawbacks from accelerometers and gyroscopes to characterize tremor movements. Additionally, this approach would benefit understanding movement synergies and tremor propagation.

### Movement prediction with video recordings

Marker-based motion capture uses optical 3D motion analysis systems to track reflective markers placed strategically on the body during movement analysis. It uses infrared cameras to capture marker movement, which is then used to calculate various spatiotemporal, kinematic, and kinetic gait parameters through software calculations [256]. In particular, Deutschl et al. [257] used marker pose estimation to observe whether people with ET showed intention tremors by instructing the participants to grasp a target. The researchers identified the presence of intention tremors similar to that seen in MS and ataxia.

Leap motion systems use multiple cameras and infrared sensors to analyze hand motions within their field of view. While highly accurate, their range of motion is limited [258, 259]. Chen et al. [260] and Khwaounjoo et al. [261] used a leap motion sensor to quantify ET and PD postural tremor by measuring the finger tremor amplitude and frequency. Although their results were less accurate than using IMUs, they showed a strong correlation with respect to them; they localized the best positions for tremor identification and achieved high accuracy at lower frequencies.

Markerless pose estimation is a new technique used to estimate the position and movement of human body joints without using physical markers. Using standard video, it utilizes computer vision and machine learning algorithms to analyze movement in real-time. The technique involves detecting and recognizing key body landmarks, constructing a skeletal model, and estimating joint position and movement over time. Markerless pose estimation software is user-friendly and flexible. Still, it has limitations, including lower accuracy than marker-based systems, difficulty tracking occluded or partially visible body parts, and sensitivity to environmental factors. Nonetheless, ongoing advances in computer vision and machine learning are enhancing the accuracy and robustness of these techniques [262–267], making them potentially valuable for tremor characterization—for example, Park et al. [15] utilized Mediapipe [268] to analyze its feasibility in telemedicine for PD. Although the study involved healthy subjects, the findings suggested that movement tracking accuracy was hindered by poor video quality. Nevertheless, the researchers proposed that the software could be effectively utilized with better video setup and equipment. Furthermore, Pang et al. [269] used OpenPose [270], a real-time body pose estimation library using deep learning, to successfully track tremors and bradykinesia in PD using DWT to detect finger motion changes in the frequency domain.

In summary, marker-based estimation technologies capture tremors, but their setup and costs limit their evaluation in large patient cohorts or clinical practice. However, with advancements in computer vision based on deep learning algorithms, markerless pose estimators have the potential to become widely adopted for easy tremor analysis using simple setups such as phone cameras.

## Conclusions: future avenues to assess intention tremor

Of all the collected studies, 52 (21% of the total) assessed intention tremor tasks. Furthermore, 37% of these studies [36, 37, 56, 65, 66, 71, 84, 115, 122, 124, 183, 193, 210, 241, 251–253, 257, 265] (less than 8% from all studies) focus on pwMS, ataxia, or cerebellar disease, who tend to exhibit intention tremor more clearly. The findings indicate that assessment technologies measuring intention tremor should design tasks that elicit intention tremor and involve individuals who exhibit relevant symptoms.

Although digitized drawings have been examined in people with intention tremor [14, 55, 56, 58, 65, 66], further comparison with other intention tremor tasks is needed, such as the SARA scale and the FTN or finger chase tasks. Moreover, the effectiveness of digitized drawings in eliciting intention tremor and their association with task-specific tremors require more investigation.

Regarding physiological sensors, EMG has been used in pwMS [84, 95]. Still, only one study has explored its application in intention tremor [84], yet their findings did not provide conclusive evidence concerning the relationship between accelerometry and EMG. The understanding of muscle activity in intention tremor remains incomplete, necessitating a more comprehensive analysis. For instance, conducting tasks specifically designed to elicit intention tremor in individuals with cerebellar pathology would facilitate an in-depth investigation of motor conduction times and activation patterns [62].

EEG could help to differentiate movement intention from tremor, as previously suggested by Gallego and Ibáñez et al. [98, 238] in their analysis of tremor in ET. Examining patients' brain activity with intention tremors may shed light on how cortical or cerebellar activities change during motor control tasks. From computational neuroanatomy and neuroimaging studies, the premotor, primary motor, parietal regions of the cortex, and cerebellum are believed to be involved in motor control [271] and tremorous movements [101, 272]. Assessing cerebellar activity during motor control and intention tremor tasks could be valuable, especially for patients with cerebellar pathology [107, 273, 274]. For example, recent studies observed heightened cerebellar activity through cerebellar EEG recordings of ET patients [105] with only one study, to the best of the authors’ knowledge, using an intention tremor task [106]. Additionally, the interaction between the motor, parietal, and cerebellar regions could be analyzed during motor execution and intention tremor tasks. A past study investigated the functional interaction (using EEG modular functional connectivity) of the somatomotor system and higher-order processing systems during a motor task [275].

Motion capture algorithms could be one of the best ways to assess intention tremors due to their easy integration with wearable technologies for intervention, such as tremor-damping exoskeletons. The valuable research conducted by Morgan et al. [115] and Deuschl et al. [257], investigating intention tremor during activities that induce this type of tremor, can now be easily replicated using markerless pose estimation software, as done by Pang et al. in PD [269]. On the other hand, IMU sensors have become practical and effective for tremor detection but require sensor fusion algorithms and signal processing techniques for reliable analysis [90, 183, 242]. Another study was performed by Carpinella et al. [183] effectively employed the combined capabilities of EMD and HHT to accurately detect minute variations in intention tremor tasks. They accomplished automatic classification and distinction between HS and pwMS and detected subtle tremors from voluntary movement in MS. Furthermore, Tran et al. [251, 252] used ballistic tracking (an intention tremor task analogous to the finger chase test) with an IMU and a Kinect camera to distinguish between ataxia and HS successfully. These outcomes present promising prospects for the automated detection and assessment of intention tremors. In addition to facilitating such analysis, this technique could also provide valuable insight into developing intention detection algorithms for individuals with neurological conditions such as pwMS, thereby enabling wearable technologies to function not only as assessment tools but also as sensors for interventions and assistive technologies in daily life.

This review examined the utilization of sensor technology in evaluating tremors across various neurological conditions. Some limitations of our review include manuscripts with unclear terminology related to tremor, e.g., studies not differentiating between the different types of kinetic tremor, and studies with imprecise methodology, especially on sensor fusion with IMUs. Nevertheless, in this review, we tried to the best of our abilities to systematically infer those missing fields using the information in other parts of the manuscripts, e.g., experimental protocol and patient population, to infer tremor type and results and conclusions to infer sensor fusion modalities.

**Fig. 3:**
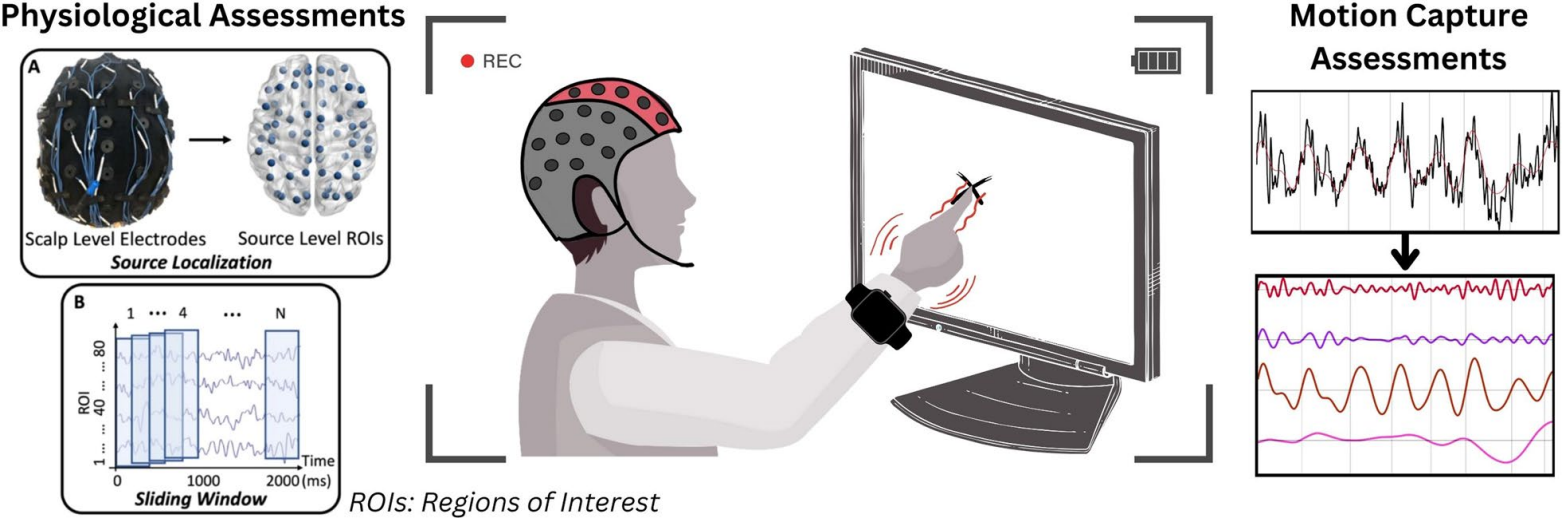
Intention tremor can be further studied through technology and specialized tasks, which isolate and amplify it. EMG and EEG provide insights into source localization and connectivity. Motion capture technologies and algorithms such as EMD reveal details about voluntary and involuntary actions. The figure is adapted from [276] and used under granted copyright by CCC RightsLink

While most research has focused on assessing tremor in PD and ET, intentional tremors observed in patients with lesions in the cerebellum could be better understood. This challenge can be approached by targeting intention tremors and leveraging existing technology (see Fig. 3). First and foremost, a technical contribution is needed to make better intention tremor assessments beyond the current tests. Furthermore, analyzing muscle activation and brain activity through EMG and EEG can provide insights into the underlying causes of intentional tremors. Regarding motion capture, it is crucial to optimize IMUs through sensor fusion algorithms that utilize the strengths of each sensor (accelerometer, gyroscope, magnetometer) to obtain an accurate limb position to extract tremorous movements using time–frequency analysis.

Additionally, using markerless pose estimation would offer a more straightforward and flexible means of capturing data without requiring specialized equipment, enabling assessments to be conducted on more subjects exhibiting intention tremors, for example, at home. Distinguishing between voluntary and involuntary movement remains a challenge for the technologies discussed. Therefore, it is essential to use and further develop signal processing techniques that focus on separating different movement components, such as EMD or DWT, to enhance the detection of the distinct aspects of tremorous movements, their onset, and their differentiation from voluntary movements.

### Supplementary Information

**Additional file 1.** Studies using tremor assessment technologies classified according to its type. This additional file is a table including all studies considered in this review. The table categorizes the studies into assessment type, number and type of patients, technology used, method, purpose, and year.

**Additional file 2.** PRISMA checklist for scoping reviews. This checklist structures the reporting items of our scoping review by providing the page number where each section can be found.

**Additional file 3.** Results of literature search and data extraction. This flow diagram shows the number of sources of evidence screened and assessed for eligibility and the number of studies excluded at each stage of the data extraction process.

**Additional file 4.** Database of selected studies. This table shows the database of the selected studies. It provides additional information than Additional file 1, such as the type of IMU sensors, and disseminates the data to automatically analyze it.

**Additional file 5.** Database search strings. This text document contains the search strings used in PubMed and Scopus for data retrieval.

## Data Availability

The datasets supporting the conclusions of this article are included within the article and its additional files.

## Abbreviations

ET: Essential tremor
PD: Parkinson’s disease
MS: Multiple sclerosis
HS: Healthy subjects
ADLs: Activities of daily living
FTMRS: Fahn-Tolosa-Marin Tremor Scale
TETRAS: Essential Tremor Rating Assessment Scale
SARA: Scale for the Assessment and Rating of Ataxia
FTN: Finger to nose test
ARAT: Action Research Arm Test
9HPT: 9 Hole Peg Test
BBT: Box and Blocks Test
FT: Finger tapping
PRISMA: Preferred Reporting Items for Systematic Reviews and Meta-Analyses
fMRI: Functional magnetic resonance
MEG: Magnetoencephalography
FES: Functional electrical stimulation
EMG: Electromyography
EEG: Electroencephalogram
FFT: Fast Fourier transform
PSD: Power spectral density
DWT: Discrete wavelet transform
HHT: Hilbert-Huang transform
IMF: Intrinsic model functions
EMD: Empirical mode decomposition
VPIT: Virtual Peg Insertion Test
pwMS: People with MS
MMG: Mechanomyography
PH: Physiological tremor
ART: Age-related tremors
TMS: Transcranial magnetic stimulation
MSA-C: Multiple system atrophy cerebellar subtype
IMU: Inertial measurement units
EKF: Extended Kalman filter
IAS: Institute for Advanced Study
WFLC: Weighted frequency Fourier linear combiner
SVM: Support vector machine
CNN: Convolutional neural network
