# Supplementary material for: Upper limb intention tremor assessment: opportunities and challenges in wearable technology
Source: J Neuroeng Rehabil. 2024 Jan 13;21:8. doi: 10.1186/s12984-023-01302-9 (PMC10787996; doi:10.1186/s12984-023-01302-9)
Supplement: Supplementary file 1 — Additional file 1. Studies using tremor assessment technologies classified according to its type. This additional file is a table including all studies considered in this review. The table categorizes the studies into assessment type, number and type of patients, technology used, method, purpose, and year. [file 12984_2023_1302_MOESM1_ESM.docx]

**Additional File 1** Studies using tremor assessment technologies classified according to its type.

| Author | Symptom | Subjects & Conditions | Technology | Method | Purpose | Year |
| --- | --- | --- | --- | --- | --- | --- |
| Tasks and Tools – Manipulanda | | | | | | |
| Hacisalihzade et al. ^77^ | Intention tremor | 11 PD 83 HS | Potentiometer tracking device | Participants perform tracking tasks in manipulandum | Measure PD symptoms during tracking test | 1988 |
| Aisen et al. ^3^ | Postural intention tremor | 18 MS 8 HS | Electromechanical tremor scoring device (TSD) | Participants use manipulandum in postural and following trajectory activities | Assess MS tremor using manipulandum | 1989 |
| Beuter et al.^19^ | Rest, intention tremor | 11 PD 11 HS | Laser Matsuhita Electric Works | Participants use laser system on fingertop during activities that elicit tremor | Evaluate the use of laser systems to quantify tremor | 1994 |
| Norman et al. ^167^ | Postural tremor | 6 PD 8 HS | Laser Matsuhita Electric Works | Participants use laser system on finger during activities that elicit tremor | Evaluate the use of laser systems versus EMG and accelerometer to quantify tremor | 1999 |
| Feys et al.^63^ | Intention Tremor | 16 MS 16 HS | Buttons attached to goals, Minibird electromagnetic sensor, ASL 4100 eye tracker | Participants grasp and move a manipulanda between two points (home and goal) in different eye movement conditions | Examine the characteristics of intention tremor in motor performance and eye movements during rapid goal-directed movements | 2003 |
| Feys et al.^62^ | Intention tremor | 16 MS 16 HS | Self-built aiming task table | Eye tracking and magnetic sensors measure movement performance while participants execute aiming tasks | Examine the eye and hand movement characteristics of intention tremor during rapid goal-directed movements | 2003 |
| Papapetropoulos et al. ^175,176^ | Rest, postural, simple kinetic tremor | 44 PD 28 HS | CATSYS: portable system to record tremor | Participants use CATSYS system to measure tremor | Evaluate CATSYS in PD | 2008 |
| Goetz et al.^73^ | PD symptoms | 52 PD | AHTD: At Home Testing Device, Intel | Participants use AHTD during 6 months, AHTD includes FT, 9HPT (with 8 pegs) and wrist accelerometer | Correlate at home assessment tool with UPDRS | 2009 |
| Scanlon et al.^202^ | Rest, postural tremor | 16 PD 8 HS | Tremor Pen stylus, CATSYS | Participants used the pen by attaching it to their hand during activities that trigger tremors | Analyze capability of accelerometer in pen to assess upper and lower limb tremor | 2013 |
| Maldonado-Naranjo et al.^147^ | Rest, postural, simple kinetic tremor | 21 PD | Stylus pen,  Cleveland Clinic Stulus | Participants use pen to perform activities that elicit tremor | Evaluate sensory pen to detect tremors in PD | 2019 |
| Oliveira et al.^168^ | Postural tremor | 5 PD 5 HS | PS25454, Plessey semiconductors | Participants position hand in front of capacitive sensors | Evaluate the use of noncontact capacitive sensors to detect postural tremor | 2019 |
| Zajki-Zechmeister et al.^243^ | Rest, Postural Tremor | 16 ET 14 PD | TREM - Tremitas GmbH | Participants use the TREM Pen in two different settings: first, by holding it, and second, by attaching it to their hand during activities that trigger tremors | Capability of TREM to quantify tremor and its correlation with UPDRS and TETRAS | 2020 |
| Ferenčík et al.^59^ | Simple kinetic tremor | 7 PD 36 HS | Rehapiano based on strain gauges | Participants use Rehapiano by applying force with fingers | Verify the capability of using Rehapiano to detect action tremor | 2020 |
| Júnior et al. ^100^ | Task specific tremor | 20 HS 20 PD | Pen with embedded IMU MPU6050 | Participants use intelligent pen while drawing Archimedes spirals and writing | Evaluate effectiveness of quantifying writing tremor in PD using a pen | 2020 |
| Kanzler et al. ^101,102^ | Sensorimotor Hand Dysfunction | 11 MS, 23 AT | VPIT and OMI Haptic Interface | Machine Learning in digital health metrics of VPIT | Prediction of response to interventions | 2021 |
| Kim et al ^110^ | Simple kinetic, task specific, intention tremor | 11 ET 3 HS | 3D gyration mouse GM; AirMouse Go Plus | Participants perform Archimedes spirals, navigation task, center out task and tapping task using a 3D mouse | Quantify tremor using a 3D mouse and activities that elicit tremor | 2022 |
| Toffoli et al. ^223^ | Task specific tremor | 29 PD 29 HS | Smart Ink Pen^143^ | Participants trace an Archimedes spiral on a sheet of paper using the smart ink pen | Archimedes spirals analysis using smart pen with an embedded load cell and tri-axial accelerometer and gyroscope | 2023 |
| Tasks and Tools – Digitized Drawing | | | | | | |
| Elble et al. ^53,55^ | Task-specific Tremor | 87 ET | IBM-compatible digitizing tablet | Writing cursive "e"s and "l"s, and Archimedes spirals versus linear acceleration measurements using accelerometer | Quantitative assessment of writing tremor | 1996 |
| Riviere et al.^194^ | Rest task specific tremor | 1 ET 1 PD | SummaSketch, Summagraphics Seymour | Participants use tablet to draw Archimedes spirals and rest hand on it while holding pen | Assessing weighted frequency Fourier linear combiner (WFLC) to detect tremors | 1997 |
| Pullman^188^ | Task-specific tremor | 62 PD | Digitizing tablet, Kurta Corporation | Participants draw Archimedes spirals on a tablet | Analyze digitized Archimedes spirals in PD | 1998 |
| Erasmus et al.^57^ | Tremor and other upper limb ataxic symptoms | 342 MS, 140 HS | KYE Genius New Sketch Tablet | Participants draw an '8' on a tablet | Quantification of ataxic symptoms | 2001 |
| Feys et al.^64^ | Intention Tremor | 30 MS 15 HS | Easypen G6, Netway Components Limited | Participants draw a circular and squared Archimedes spirals in a tablet | Vailidity and reliability of digitized Archimedes spirals to distinguish MS tremor | 2007 |
| Stanley et al. ^213^ | Task-specific tremor | 9 PD 40 HS | Intuos 2, Wacom Inc | Participants draw 20 Archimedes spirals on tablet | Detect changes not detectable by UPDRS using digitized Archimedes spirals | 2010 |
| Haubenberger et al. ^79^ | Task-specific tremor | 9 ET | Wacom Digitizing Tablet | Participants draw an Archimedes spiral in a tablet | Rating tremor compared to standard clinical tests | 2011 |
| Aghanavesi et al.^2^ | Simple Kinetic, Task-specific tremor | 19 PD 22 HS | - | Participants alternate tapping two squares as fast as possible (FT) and draw Archimedes spirals on smartphone | Quantify and characterize dexterity in PD compared to UPDRS-III | 2017 |
| Legrand et al.^124^ | Task specific tremor | 13 ET | Wacom, Bamboo Fun Medium Pen and Touch | Participants draw Archimedes spirals on tablet | Propose new methods to rate Archimedes spirals | 2017 |
| Elble et al.^54^ | Task-specific tremor | 18 ET | Wacom Intuos 3 | Participants draw Archimedes spirals on tablet. Amplitude is analyzed | Compare Archimedes spirals to FTMRS in ET | 2017 |
| DelMastro et al.^48^ | Task-specific and Intention Tremor | 258 MS | Wacom Digitizing Tablet | Participants draw an Archimedes spirals in a tablet | Compare the sensibility of digitized versus manual Archimedes spirals test | 2018 |
| Zham et al.^245^ | Task-specific Tremor | 31 PD 31 HS | Wacom A3 digital tablet | Participants write letters and draw Archimedes spirals on tablet | Determine which features are better to distinguish PD and HS in Archimedes spirals drawings | 2018 |
| Szumilas et al. ^217^ | Task specific tremor | 64 PD | Untuous 2, XD-0608 Wacom | Participants trace a given template of a circle on a digitized tablet | Obtain tremor scoring equivalent to score trained by clinicians | 2019 |
| Ferleger et al. ^60^ | Task specific tremor | 3 ET 3 HS | Consumer tablet and digital pen | Participants draw Archimedes spirals and a line on commercial tablet with stylus | Remote diagnosis of people with ET using digitized drawings | 2020 |
| Creagh et al. ^43^ | MS upper limb impairment, Intention Tremor | 71 MS, 22 HS | Floodlight App | Participants draw six different shapes (DaS and Archimedes spirals) in an app | Reliably predict 9HPT times with DaS test | 2020 |
| Sisti et al. ^207^ | Task specific tremor | 16 PD 15 HS | iPad | Participants performed Archimedes spirals in iPad and Wacom tablet | Compare Archimedes spirals using iPad and Wacom tablets | 2021 |
| Graves et al. ^75^ | MS upper limb impairment | 69 MS, 18 HS | Floodlight App | Participants draw six different shapes (DaS and Archimedes spirals) in an app | Correlation with standard clinical tests (including 9HPT), fMRI, and capacity to differentiate pwMS from HS | 2022 |
| Lipsmeier et al. ^134^ | PD symptoms | 316 PD | Roche PD Mobile App | Participants use Roche PD app where they perform different activities such as DaS, as well as passive monitoring through smartwatch and smartphone | Monitor early PD symptoms | 2022 |
| Montalban et al. ^156^ | MS upper limb impairment | 76 MS 25 HS | Floodlight App | Participants draw six different shapes (DaS and Archimedes spirals) and tap random placed objects as quick as possible (FT) in an app | Correlation with 9HPT | 2022 |
| Messan et al. ^152^ | MS upper limb impairment | 89 MS 22 HS | Google Pixel 2/XL - NeuFun-TS App | Tracing with a finger 3 Archimedes spirals with different lengths | Intraindividual variation and reproducibility of smartphone-based spiral drawing | 2022 |
| Lipsmeier et al. ^132^ | HD upper limb impairment | 199 HD 20 HS | Smartphone (Galaxy J7; Samsung) | Participants draw diagonal lines, square, circle, figure of eight, or spiral shapes with the index finger, and tap one buttons on the screen as fast and regularly as possible | Differentiate patients with HD from controls | 2022 |
| Wang et al. ^234^ | Task-specific tremor | 50 ET 40 HS | Scanned paper drawn images | CNN applied to paper drawn Archimedes spirals without template | Automatic ET classification and feature extraction | 2023 |
| Tasks and Tools - Other Task/Tool based tests | | | | | | |
| Zhan et al. ^246^ | Simple kinetic tremor | 152 PD 17 HS | HopkinsPD | Participants perform FT and other motor tasks in smartphone | Develop an objective measure for PD | 2018 |
| Boukhvalova et al. ^23^ | MS upper limb impairment | 112 MS 15 HS | Smartphone (Google Pixel XL) | Participants tilt a smartphone to make a free-rolling ball stay in the center of the screen | Correlate accelerometer data during game with pwMS reaction time, cerebellar function, and proprioception | 2019 |
| Kuosmanen et al. ^117^ ^118^ | Task-specific tremor | 13 PD | STOP app | Participants tilt a smartphone to keep a ball inside a circle in the center of the screen | Monitor and assess tremor in PD before/after medication | 2020 |
| Gulde et al. ^76^ | MS upper limb impairment | 12 MS | Lumia 550 smartphone | Participants perform FT on a smartphone | Correlate changes in FT with central motor conduction time and 9HPT | 2022 |
| Wilkins et al. ^237^ | PD symptoms | 96 PD 42 HS | Digitography Device | Participants use a digitography device to perform FT | Quantify FT to measure motor impairment in PD and correlate to UPDRS | 2022 |
| Physiological sensors – EMG | | | | | | |
| Bacher et al. ^10^ | PD symptoms | 1 PD | EMG electrodes and Oxford PMD12 | Participant uses EMG on forearm muscles for 24 hours | Asssess the feasibility of using EMG for PD tremor detection | 1989 |
| Deuschl et al. ^50^ ^222^ | Rest Tremor | 21 RSD | - | Record movement with EMG and accelerometer when subjects sit with arms supported and hands outstretched | Find best signal processing method using FFT and PSD to detect tremors | 1996 |
| Spieker et al. ^211,212^ | PD symptoms | 27 PD 19 ET | OMNIlog; CSM, Germany | Participants perform UPDRS during EMG measurements | Correlate EMG readings with clinical parameters for tremor quantification | 1997 |
| Foerster et al. ^65^ | Rest, postural tremor | 27 PD | - | Participants use accelerometer and EMG in activities that elicit tremor | Examine amplitude and frequency of tremor in PD | 1999 |
| Milanov ^153^ | Rest postural intention tremor | 110 PD 220 ET 22 AT 173 other | - | Participants use EMG sensors on limbs during different motor activities | Differentiate tremor based on EMG | 2001 |
| Brennan et al. ^26^ | Postural, Intention Tremor | 50 ET | - | Participants use accelerometer and EMG while performing arm extension and FTN | Evaluate if ET symptoms are primarily kinetic or postural | 2002 |
| Cohen et al. ^40^ | Rest tremor | 5 ET | - | Participants used EMG and accelerometer during activities that elicit tremor | Determine prevalence of rest tremor in ET | 2003 |
| Zeuner et al. ^244^ | Simple kinetic tremor | 11 ET 12 PD 6 other | Kistler Instrument Corp | Participants use accelerometer and EMG during FT | Differentiate psychogenic tremor from ET and PD | 2003 |
| Piboolnurak et al. ^183^ | Rest postural intention tremor | 15 ET 22 PD 21 HS 34 other | - | Participants use accelerometers and EMG while performing activities that elicit tremor | Quantify and analyze psychogenic tremor | 2005 |
| Wang et al. ^233^ | Rest tremor | 1 PD | EMG H27P, Kendall-LTP, USA | Participants use EMG over several episodes of transient resting tremor | Analyze tremor changes during intermittent resting tremor | 2005 |
| Breit et al. ^25^ | PD and ET upper limb impairment | 26 PD 19 ET | - | Participants use EMG during their daily activities for 24 hours | Long-term EMG automated analysis to differentiate PD from ET | 2008 |
| Nisticò et al. ^164^ | Rest Tremor | 15 ET 15 PD | - | EMG recorded with the arm flexed at 90$^\circ$, fully supported against gravity | Differentiate subjects with ET and PD. Activation in antagonist muscles is in sync in ET and alternating in PD | 2010 |
| Basu et al. ^13^ | Rest postural simple kinetic tremor | 4 PD 4 ET | EMG: delsys, acc: Coulbourn type V94-41 | Participants use EMG and accelerometer during activities that elicit tremor | Predict tremor onset using EMG and accelerometer | 2013 |
| Roy et al. ^195^ | PD symptoms | 19 PD 4 HS | - | Participants use sensors during continuous monitoring of symptoms | Evaluate the use of EMG with accelerometers for continuous monitoring of PD symptoms | 2013 |
| Hossen et al. ^86^ ^90^ | Postural Tremor | 41 ET 39 PD | - | Participants extended their hands and fingers in a position against gravity | Use 3 different signal processing methods to differentiate between ET and PD | 2014 |
| Cole et al. ^41^ | PD symptoms | 8 PD 8 HS | EMG and Acc: Delsys Inc. | Participants use smartwatch and EMG during ADLs in an apartment-like environment | Evaluate machine learning algorithms to track tremor and dyskinesia in PD | 2014 |
| Ayache et al. ^8^ | Postural tremor | 8 ET 8 physiological tremor | EMG: Natus-Dantec, Accelerometer: TREM0000, Neuroservices, Evry-Lisses | Participants use EMG and accelerometer during position against gravity | Use HHT to differentiate between ET and physiological tremor | 2014 |
| Ayache et al. ^9^ | Postural, intention tremor | 32 MS | EMG: Natus-Dantec, Accelerometer: TREM0000, Neuroservices | Participants use accelerometer and EMG during a position against gravity and FTN | Correlate accelerometer and EMG measurements and detect tremor in pwMS with and without visible tremor | 2015 |
| Ghassemi et al. ^71^ | Rest and Postural Tremor | 11 ET 13 PD | EMG - Schwarzer Topas Natus | Participants perform tasks eliciting postural and rest tremor while accelerometer readings and EMG are recorded | SVM classification between ET and PD | 2016 |
| Boroojerdi et al. ^21^ | PD upper limb impairment | 25 PD | NIMBLE patch | Participants use a sensor with an embedded accelerometer and EMG while performing UPDRS-III | Observe sensor correlations to PD symptoms | 2019 |
| Hossen et al. ^88^ | Postural tremor | 41 ET 39 PD 19 PH | - | Participants outstretch arms while using a 2 g accelerometer and EMG in flexor and extensor muscles | Differentiate physiological tremors from PH, PD, and ET | 2020 |
| Gorbunov et al. ^74^ | Rest tremor | 11 ET | - | Participants use accelerometer on wrist during sleep | Quantify night tremors in ET | 2020 |
| Vescio et al. ^232^ | Rest tremor | 7 ET 14 PD | EMG - Myoware | Record EMG with arm flexed at 90$^\circ$, with full gravity support | Evaluate $\mu$EMG system capabilities to detect rest tremor and differentiate between ET and PD | 2021 |
| Hossen et al.^87^ | Postural tremor | 41 ET 39 PD 40 MS | - | Participants outstretch arms while using a 2 g accelerometer and EMG in flexor and extensor muscles | Differentiate MS tremors from PT and ET and correlate measure to FTMRS and UPDRS scale | 2022 |
| Nisticò et al. ^165^ | Rest tremor | 90 PD 24 ET | - | EMG recordings are done in three different rest positions | Compare rest tremor features in different positions in PD and ET | 2022 |
| Lin et al. ^131^ | Rest, postural, intention tremor | 40 PD | IMU: Shimmer3 and EMG | Participants perform activities that elicit tremor while using IMU and EMG | Extract tremor signals using EMG and IMUs | 2023 |
| Physiological sensors – MMG | | | | | | |
| Angeles et al. ^5^ | Rest, Postural, Intention tremor | 7 PD | MMG: Biomechatronics Lab, Imperial College | Participants use a force sensor, IMU, and MMG sensor in activities that elicit tremor | Evaluate the use of MMG, EMG, and IMUs to detect deep brain stimulation differences in PD | 2017 |
| Huo et al. ^92^ | Rest, Postural, Intention tremor | 23 PD 10 HS | IMU: STM, MMG: Biomechatronics Lab, Imperial College | Participants use a force sensor, IMU, and MMG sensor in activities that elicit tremor | Evaluate the use of MMG, EMG, and IMUs with ML to detect PD symptoms and correlate to UPDRS | 2020 |
| Physiological sensors – EEG | | | | | | |
| Verleger et al. ^231^ | AT symptoms in force exertion | 12 AT 10 HS | Nihon-Kohden 4421 | Measured force of participants in holding a key is correlated with ERP analysis | Findings suggest cerebellum is less active in fine coordination than in motor planning compared to the motor cortex | 1999 |
| Muthuraman et al. ^161^ | Postural tremor | 10 ET 10 PD 10 HS | Neuroscan Herndon, USA | EEG and EMG is recorded in participants while holding a position against gravity for 1 to 4 mins | Source localization of pathological tremor and voluntary mimicked tremor. Findings suggest that both tremors involve the cerebellum and other motor regions | 2012 |
| Ibáñez et al. ^93^ | Intention tremor | 4 ET 6 HS | gUSBamp, g.Tec GmbH | Participants use EEG cap and IMUs on hands during reaching movements | Develop an EEG online detector of intentional movement | 2013 |
| Muthuraman et al. ^160^ | Postural, simple kinetic tremor | 20 ET 10 ART | - | Participants perform slow kinetic movements and hold positions during EEG and EMG measurement | Source localization and differences between ART and ET. The results suggest that people with ET have strong cerebellar involvement compared to ART | 2015 |
| Pedrosa et al. ^180^ | Rest, postural tremor | 20 ET 20 HS | Easy-Cap GmbH, Germany | EEG and EMG is recorded while participants are at rest and holding a posture before and 30 minutes after 330 ml (4,8\%) of alcohol intake. HS are asked to mimic tremor | Source localization and amplitude modulation between ET and HS tremors. The results suggest a higher involvement of the supplementary motor area in ET, and cerebellar coupling with movement after alcohol intake | 2017 |
| Muthuraman et al. ^162^ | Postural tremor | 34 ET 34 PD 34 HS | EGI system | Participants were asked to hold a position against gravity for 2 min while EEG and EMG was recorded. For HS, participants were asked to mimic tremor | Localize topography of cerebellar acitivty in PD, ET and mimicked tremor. In mimicked tremor funcitonal connection is between premotor cortex and cerebellum, whereas in ET and PD is between sensorimotor cortex and cerebellum | 2018 |
| Aoh et al. ^6^ | AT in simple kinetic movements | 15 AT 15 HS | NeuroScan SynAmps, Neurosoft, Inc | Participants lift the right palm every 6 seconds, 60 times during EEG and EMG recordings | ERD/ERS analysis of motor tasks in AT suggesting that the cerebellum is mostly involved in preparing and executing the movement | 2019 |
| Song et al. ^210^ | AT rest position | 50 MSA-C 25 HS | EEG amplifier Yunshen Ltd, 2 electrodes cap Greentek Ltd | Participants stay relaxed for 20 minutes during TMS while EEG is measured at the same time | Measure improvement of TMS on cerebello-frontal connectivity and SARA | 2020 |
| Pan et al. ^173^ | ET symptoms | 20 ET 20 HS | - | EEG measured in participants is analyzed based on hypotheses from mouse tremor models and postmortem ET cerebellum reflecting synaptic pruning deficits of climbing fiber to Purkinje cell | Correlate excessive cerebellar oscillations in ET with tremor severity | 2020 |
| Wong et al. ^239^ | Intention tremor | 40 ET 20 HS | Cervello 32, Blackrock Microsystem Inc | Cerebellar EEG is measured while participants perform a similar activity to FTN | Correlate excessive cerebellar oscillations in ET with tremor severity. Finding suggests that severity is related only with familial ET in contrast with sporadic ET | 2022 |
| Bosch et al. ^22^ | Rest tremor | 75 PD 39 HS | 64-ch EEG cap | Cerebellar EEG is measured in participants during rest state | Examine cerebellar activity in PD versus HS. Findings suggest an abnormal and increased theta freq band (4-7Hz) oscillations in PD | 2022 |
| Motion Capture Sensors - Accelerometers, Gyroscopes and IMUs | | | | | | |
| Morgan et al. ^157^ | Intention tremor | 10 MS 5 ET 1 PD 2 AT 12 Other | Acc: M.E.M. Electro Mechanisms Ltd | Participants use accelerometer on top of the hand while performing a task similar to FTN | Analyze intention tremor using accelerometer | 1975 |
| Cleeves et al. ^39^ | Rest Tremor | 36 ET | Endevco 7625-10 | Participants use accelerometer on dorsal face of hand during rest tremor activities | Examine tremor amplitude with accelerometer in ET | 1987 |
| Van Someren et al. ^228^ | Activities of daily living | 4 PD 1 ET 10 HS 3 other | Acc: ENDEVCO Picochip | Participants use wrist-worn accelerometer in daily life for 24 hours | Monitor tremor before and after thalamotomy | 1993 |
| Kulisevsky et al. ^116^ | Rest, Postural Tremor | 20 PD | Accelerometer Grass SPA | Participants use accelerometer on both index fingers with and without medication during activities that elicit tremor | Quantify the efficacy of medication (levodopa) in PD tremor | 1995 |
| VanSomeren et al. ^229^ | PD upper limb impairment | - | ENDEVCO Picochip | Participants use accelerometer on the wrist while performing UPDRS | Validate the use of accelerometers to assess tremors in PD | 1998 |
| Jankovic et al. ^97^ | Rest Postural tremor | 27 ET 18 PD | - | Participants use accelerometer on hand during activities that elicit tremor | Compare re-emergent tremor in PD compared to ET | 1999 |
| Matsumoto et al. ^149^ | Postural tremor | 30 ET | Acc: Grass SPA1, Astromed Inc. | Participants use accelerometer on fingertip during activities that elicit tremor | Quantify tremor in ET using accelerometer | 1999 |
| Su et al. ^241^ | Rest tremor | 1 PD 1 HS | 3D electromagnetic tracking system | Participants use sensory glove in rest position | Evaluate the use of 3D sensor glove to detect tremor | 2003 |
| Hoff et al. ^85^ | PD symptoms | 15 PD | IC sensors, Measurement Specialities Inc. | Participants use accelerometers continuosly | Examine the accuracy of objective ambulatory accelerometry in detecting motor complications in PDaccelerometer | 2004 |
| Thielgen et al. ^221^ | Rest postural tremor | 30 PD | Vitaport-2 | Participants use accelerometers for 24 hours during daily life. Readings are compared to rest and postural tremor measurements | Examine constant monitoring of PD symptoms with accelerometers | 2004 |
| Caligiuri et al. ^30^ | Rest, postural tremor | 60 PD 35 ET 26 HS 147 other | Tremometer with accelerometer: ADXL-210 | Participants use tremometer while performing activities that elicit tremor | Evaluate Tremometer to detect tremors | 2004 |
| Rocon de Lima et al. ^130^ | Rest, Postural, Simple kinetic, Task-specific tremor | 21 ET 3 PD 4 AT 3 other | - | Analyze and decompose gyroscope signals into tremorous and voluntary activity using EMD and HS | Analyze and decompose signals into tremorous and voluntary activity using EMD and HS | 2006 |
| Keijsers et al. ^105^ | PD symptoms | 23 PD | ADXL202 Analog Devices | Participants use accelerometers for 3 hours during ADLs | Distinguish between on and off states of people with PD | 2006 |
| Smeja et al. ^208^ | Rest postural tremor | 25 PD | - | Participants use accelerometer during postural and rest tremor tasks as well as 24 hoursb ambulatory monitoring | Quantify tremor and posture continuously in PD | 2006 |
| Salarian et al. ^198^ | PD symptoms | 10 PD 10 HS | Gyro: Murata ENC-03J | Participants use sensors during ADLs | Evaluate algorithm to detect and quantify tremor using gyroscopes | 2007 |
| Shaikh et al. ^204^ | Rest postural tremor | 35 ET 19 cervical dystonia | - | Participants use accelerometer on finger during activities that elicit tremor | Differentiate tremor in cervical dystonia from ET | 2008 |
| Giuffrida et al. ^72^ | Rest, Postural, Simple kinetic tremor | 60 PD | Kinesia sensors, CleveMed | Participants use sensor on the finger while performing activities that elicit tremor | Evaluate Kinesia system to automate assessment of tremor in PD | 2009 |
| Patel et al. ^178^ | Rest, Simple Kinetic, Intention tremor | 12 PD | SHIMMER system, Accelerometer Freescale MMA7260Q | Participants use accelerometers along arms and legs while doing UPDRS tasks that elicit tremor | Using SVM classifiers to estimate tremor severity using accelerometers | 2009 |
| Gallego et al. ^69^ | Rest, Postural, Simple kinetic, Intention tremor | 2 ET 1 PD 2 other | IMUs - Technaid S.L. | Participants use two gyroscopes on hand and forearm during tasks that elicit tremor | Real-time estimation of tremor parameters using a WFLC for frequency and KF for amplitude | 2010 |
| LeMoyne et al. ^125^ | Rest Tremor | 1 PD 1 HS | iPhone 3G | Participants use iPhone on the dorsal part of the hand in rest position | Evaluate the use of an iPhone application as a wireless accelerometer system to record and quantify tremors in PD | 2010 |
| Mostile et al. ^158^ | Postural, simple kinetic tremor | 20 ET | Kinesia sensors, CleveMed | Participants outstretch arms and perform repeatedly movements using IMU finger sensor | Evaluate correlation of Kinesia sensors and TETRAS | 2010 |
| Zwartjes et al. ^252^ | Rest postural intention tremor | 6 PD 7 HS | MT9 inertial sensors, Xsens Tech. | Participants perform UPDRS-III while using sensors | Examine motor assessment in comparison with UPDRS | 2010 |
| Heldman et al. ^84^ | Postural, Kinetic Tremor | 10 ET | Kinesia sensors, CleveMed | Participants use sensor on index finger while performing wTRS and ADLs | Classify type of tremor and severity during standardize activities and ADLs | 2011 |
| Ketteringham et al. ^106^ | Rest, Postural, Intention tremor | - | MTx - Xbus kit, Xsens Technologies | Subjects use five IMUs along their limbs while performing selected FTMTRS tests | Record intention tremor using IMUs | 2011 |
| Joundi et al. ^99^ | Rest, Postural Tremor | 2 ET 1 PD 1 MS 3 Other | iSeismo App - Iphone | Participants use iPhone and EMG attached to the tremorous limb during activities that elicit tremor | Evaluate the frequency measurement accuracy of iSeismo app compared to EMG measurements | 2011 |
| Gallego et al. ^68^ | Postural Tremor | 17 DD | EMG: OT Bioelectronica, IMU: TechMCS, Technaid SL | Participants use EMG, EEG, and IMU in a postural test | Estimate Tremor Feature for FES exoskeleton tremor suppressor | 2011 |
| Niazmand et al. ^163^ | Rest, Postural Tremor | 9 PD 3 HS | Acc: SMB380 Bosch | Participants use sensory glove with embedded accelerometers during activities that elicit tremor | Estimate PD symptoms using accelerometer | 2011 |
| Rigas et al. ^193^ | Rest postural task-specific intention tremor | 18 PD 5 HS | - | Participants perform activities that elicit tremor while wearing accelerometers | Quantify tremor and discriminate rest from postural tremor in PD | 2012 |
| Mera et al. ^151^ | Rest postural tremor | 15 PD | KinetiSense, Great Lake Neurotech | Participants use sensors while executing activities that elicit tremor | Quantify levodopa-induced dyskinesia and differentiate it from tremor in PD | 2012 |
| Synnott et al. ^216^ | Rest, postural, simple kinetic tremor | 1 PD 9 HS | Nintendo Wii remote acc: ADXL 330 | Participants play different Wii games that elicit tremor | Evaluate the use of Wii remote accelerometers to assess tremor | 2012 |
| Teskey et al. ^218^ | Intention tremor | 9 ET 30 PD 11 HS | Acc: LIS3LO6AL Gyr: XV-8100CV | Participants grab the IMU and perform pointing movements with a laser | Quantify tremor before and after treatment | 2012 |
| Daneault et al. ^46^ | Rest, postural, simple kinetic, intention tremor | 12 PD 3 ET 1 MS | Smartphone Blackberry Storm | Participants perform activities that elicit tremor | Evaluate the capabilities of a smartphone accelerometer to detect tremor versus UPDRS | 2013 |
| Hossen et al. ^89^ | Postural Tremor | 41 ET 39 PD | - | Participants extended their hands and fingers in a position against gravity | Differentiate PD from ET using FFT of the accelerometer signals | 2013 |
| Rahimi et al. ^190^ | Rest postural intention tremor | 10 PD | Electrogoniometer SG65, accelerometer Noraxon | Participants perform activities that elicit tremor while using sensors | Effect of BOoNT-A injection on motor symptoms in PD | 2013 |
| Iwasaki et al. ^96^ | Rest, postural, intention tremor | 7 PD 1 ET 4 Other 10 HS | Acc: PhidgetSpatial 1056 | Partcipants use accelerometer on upper limbs during activites that elicit tremor | Assess the use of accelerometers to measure neurological motor impairments | 2013 |
| Lambrecht et al. ^122^ | Intention Tremor | 6 ET | IMUs - Tech MCS, Technaid S.L. | Participants perform finger-to-nose test while using 4 IMUs in upper limbs | Generate context-aware algorithms to detect sensor-location and track tremors using orientation data | 2014 |
| Wile et al. ^236^ | Rest, Postural Tremor | 14 ET 15 PD | ENTRAN EGAS accelerometer and WIMM One Smartwatch | Participants rest and outstretched their hands for 60 seconds with a smartwatch and an analog accelerometer | Evaluate the effectiveness of accelerometers in smartwatches vs traditional accelerometers | 2014 |
| Tzallas et al. ^227^ | Rest, Postural Tremor | 44 PD | PERFORM System, ALA-6g accelerometer, AGYRO gyroscope/accelerometer | Previous datasets are tested and in addition participants perform a wearability system test | Evaluate PERFORM system to monitor motor signals in PD | 2014 |
| Pulliam et al. ^186^ | Rest postural intention tremor | 20 ET | Kinesia HomeView, Great Lake Neurotech | Participants use sensors for up to 10 hours daily and complete standard tremor tests | Evaluate ability of motion sensors to quantify tremor during daily life | 2014 |
| Woods et al. ^240^ | Postural tremor | 14 PD 18 ET | HTC Desire, acc: BMA150 | Participants holds phone in the hand while performing activities that elicit tremor | Discriminate between postural tremor in PD and ET using smartphone | 2014 |
| Heldman et al. ^82^ | Rest postural simple kinetic tremor | 18 PD | Kinesia, Great Lake Neurotech | Participants perform activities that elicit tremor after deep brain stimulation | Determine the reliability and responsiveness of a portable kinematic system for quantifying PD motor compared to clinical ratings | 2014 |
| Bhidayasiri et al. ^20^ | Rest, postural, intention tremor | 10 ET 10 PD | - | Participants use IMU on index finger while performing activities that elicit tremor | Identify and differentiate tremor in ET and PD | 2014 |
| Carpinella et al. ^31^ | MS upper limb function | 21 MS 12 HS | IMU MTX, Xsens | Participants use IMU on wrist while performing ARAT | Correlate ARAT with IMU readings | 2014 |
| Thanawattano et al. ^220^ | Rest, postural, intention tremor | 35 PD 22 ET | - | Participants use IMU on index finger while performing activities that elicit tremor | Identify and differentiate tremor in ET and PD | 2015 |
| Pan et al. ^172^ | Rest tremor | 40 PD | App "PD Dr", smartphone accelerometer | Participants place smartphone on top of the hand to assess tremors using accelerometer | Evaluate app for PD monitoring and assessment | 2015 |
| Carpinella et al. ^32^ | Intention tremor | 20 MS 13 HS | IMU MTX, Xsens | Participants perform the FTN test with an IMU on the dorsum of the hand | Differentiate MS intention tremor from HS using Hilbiert-Huan transform | 2015 |
| Senova et al. ^203^ | Postural, simple kinetic tremor | 8 ET | iPod touch 3-ax accelerometer | Participants outstretched arms and perform repetitive movements while having an iPod fixed around their wrists | Evaluate correlation with FTMTRS score | 2015 |
| Dai et al. ^45^ | Rest, Postural, Simple Kinetic tremor | 7 PD 9 HS | MPU6050 InvenSense Inc. | Participants use an IMU on the finger tip while performing activities that elicit tremor | Correlating tremor amplitudes with neurologist judgment and electromagnetic sensor | 2015 |
| Kostikis et al. ^113^ | Rest, Postural tremor | 25 PD 25 HS | iPhone | Participants use iPhone on the dorsal part of the hand in positions that elicit tremor | Assess and quantify tremor in PD using accelerometer and gyroscope signals | 2015 |
| Bazgir et al. ^16^ | Rest, Postural tremor | - | Sony Xperia SP | Participants use phone around wrist while doing activities that elicit tremor | Use Neural Networks to classify tremors measured by accelerometer | 2015 |
| Ferreira et al. ^61^ | PD symptoms | 11 PD | SENSE-PARK System | Participants use system (accelerometer+gyroscope) for 12 weeks during their daily life | Evaluate system for long-term monitoring of PD symptoms | 2015 |
| Havlík et al.^80^ | Rest, Postural, Intention tremor | 31 MS | LIS331DLH accelerometer | Participants use accelerometer while performing activities that elicit tremor | Assess accelerometer device to quantify tremor in MS | 2015 |
| Atashzar et al. ^7^ | Rest, Postural tremor | 13 ET 14 PD | 3 DOF Accelerometer Biometrics Ltd. | Participants use sensors on upper limb while performing activities that elicit tremor | Develop an adaptive filter based on BMFLC for robotic rehabilitation architecture that reduces tremors | 2016 |
| Bermeo et al. ^18^ | Rest, Postural, Intention tremor | 3 PD 3 HS | Gyroscope MPU-6050 | Participants use sensor on dorsal side of hand and data is streamed to "Parkinson Evaluator" App | Monitor tremors in PD using mobile app | 2016 |
| Kubben et al.^115^ | ET symptoms | 3 ET | iPhone 6 | Accelerometer and Gyroscope reading from smartphone to app | Proof-of-concept of TREMOR 12 app to detect tremor in subjects with ET | 2016 |
| Fraiwan et al. ^66^ | Rest tremor | 21 PD 21 HS | Samsung Galaxy S2 | Participants use a smartphone attached to their arm while at rest for 30 seconds | Evaluate the accuracy of tremor detection using smartphone accelerometers | 2016 |
| Lee et al. ^123^ | Rest postural tremor | 114 PD | Acc: LIS3DSH STM | Participants use accelerometer on fingertip during activities that elicit tremor | Analyze tremor characteristics during rest and stress conditions in PD | 2016 |
| Koçer et al. ^112^ | Rest tremor | 35 ET 20 HS | Nintendo Wii - Wiimote | Participants held the WiiMote during activities that elicit tremor | Evaluate Hoehn and Yahr Scale using Wii accelerometer | 2016 |
| Rigas et al. ^192^ | Rest, postural tremor | 13 PD 1 HS | Acc, gyr: Microsoft Band | Participants use accelerometer and gyroscope during UPDRS and selected activities | Estimate UPDRS in home environment | 2016 |
| Surangsrirat et al. ^215^ | Rest, intention tremor | 32 PD 20 ET | - | Participants use gyroscope on index finger during activities that elicit tremor | Use support vector machine (SVM) to differentiate between PD and ET using gyroscope | 2016 |
| Contreras et al. ^42^ | Rest tremor | 12 PD | Android Wear | Participants use smartwatch at rest | Quantify PD tremors with smartwatches | 2016 |
| Bravo et al. ^24^ | Rest, postural, simple kinetic tremor | 5 PD | Acc: MPU-6050 | Participants use accelerometer during activities that elicit tremor | Analyze finger tremor with and without medication in PD | 2017 |
| Zheng et al. ^250^ | Rest, Postural, Simple Kinetic, Intention, Task-specific tremor | 8 ET | Pebble smartwatch with triaxis accelerometer | Participants perform the tasks of FTMTRS while using smartwatch | Evaluate feasibility of continuous monitoring of tremor and correlation with FTMTRS scores | 2017 |
| Jeon et al. ^98^ | Rest tremor | 85 PD | Accelerometer LIS3DSH, Gyroscope L3GD20 | Participants use sensors on finger tip while performing activities that elicit rest tremor | Predict UPDRS scores using machine learning methods on accelerometer and gyroscope signals | 2017 |
| Lima et al. ^129^ | PD upper limb impairment | 953 PD | Fox Wearable Compation App and Pebble Smartwatch | Participants used smartwatch and app 24/7 for longer than 6 weeks | Assess feasibility of wearable technology to collect data from multiple sensors in PD during ADLs | 2017 |
| Barrantes et al. ^12^ | Rest, Postural Tremor | 16 ET 17 PD 12 HS 7 Other | iPhone 5S | Participants use phone on dorsal side of the hand while performing activities that elicit tremor | Distinguish between HS, PT and ET using accelerometers | 2017 |
| Heldman et al. ^83^ | Rest Postural tremor | 18 PD | Kinesia Sensors, Great Lakes Neurotech | Participants perform motor assessments at home 1 day per week for 7 months | Assess the impact of motion sensor-based telehealth diagnostics on PD clinical care and management | 2017 |
| Locatelli et al. ^137^ | Rest postural simple kinetic tremor | 16 PD 11 ET | MuSe, acc: H3LIS331DL, gyr: ITG3701 | Participants use sensor on hand during activities that elicit tremor | Pilot study for tremor classification using wearable sensor | 2017 |
| Tsiouris et al. ^226^ | Rest postural tremor | 11 PD | PD-Manager including accelerometer and gyroscope | Participants perform ADLs and activities that elicit tremor while using the system | Estimate tremor and other PD symptoms using PD-manager | 2017 |
| Bai et al. ^11^ | Postural tremor | 13 PD 12 HS | MPU9250 - IMU | Participants use IMUs on whole body during activities that elicit tremor | Quantify PD motor symptoms | 2017 |
| Molparia et al. ^155^ | Rest, Postural Tremor | 27 ET 40 PD | Acc: LG G2 smartphone | Participants perform activities that elicit tremor while holding smartphone | Differentiate ET from PD using accelerometers and genetic information | 2018 |
| Lopez et al. ^140^ | Postural, Rest, Simple Kinetic Tremor | 28 ET | Sony Smartwatch 3 | Participants use smartwatches during tasks that elicit tremor | Correlate gyroscope readings with FTMTRS | 2018 |
| Santiago et al. ^200^ | PD symptoms | 89 PD | KinetiGraph - PKG, Movement Recording System | Participants use system for more than 6 days during daily life | Evaluate the impact of using continuous objective measurement using the KinetiGraph in the routine clinical care of PD | 2018 |
| Chan et al. ^34^ | Rest, postural, simple kinetic tremor | 38 PD | IMU: IG-500A SBG Systems | Participants use 3 IMUs along upper limb while performing activities that elicit tremor | Investigate coupled degrees of freedom movements in PD | 2018 |
| Pulliam et al. ^187^ | PD upper limb impairment | 13 PD | Kinesia Sensors, Great Lakes Neurotech | Participants performed ADLs with and without medication while using accelerometer and gyroscope on the wrist and ankle | Correlate sensor measurements of tremor, bradykinesia, and dyskinesia with UPDRS-III and ON/OFF medication | 2018 |
| Cai et al. ^29^ | Rest, postural tremor | 34 PD 14 HS | MPU6050 InvenSense Inc. | Participants use an IMU on their wrist while performing activities that elicit tremor | Correlation of IMU readings with UPDRS | 2018 |
| Sanchez-Perez et al. ^199^ | Rest tremor | 57 PD | - | Participants use an IMU on the hand while performing activities that elicit tremor | Prevent floor/ceil effect of clinical tests by quantifying tremor and using fuzzy inference to score it | 2018 |
| Lonini et al. ^139^ | PD upper limb impairment | 20 PD | IMU/EMG BioStamp RC | Participants use sensors on both hands and upper-arms while performing ADLs | Evaluate the training data/users needed to evaluate PD tremor in ADLs | 2018 |
| Delrobaei et al. ^49^ | Rest, Postural Tremor | 40 PD 22 HS | IMU: IGS-180 Synertial Ltd. | Participants use 17 IMUs while performing rest/postural tasks with and without medication (levodopa) | Evaluate correlation between UPDRS and quantified postural and rest tremor | 2018 |
| Zhang et al. ^248^ | Rest, Postural Tremor | 24 ET 26 PD | EMG and accelerometer: Biometrics Datalog | Participants use accelerometer on middle finger and EMG sensors along the forearm during rest and stretch activities | Evaluate which arm position is better to quantify tremor | 2018 |
| Bazgir et al. ^17^ | Rest, Postural Tremor | 52 PD | Sony Xperia SP | Participants use phone around wrist while doing activities that elicit tremor | Correlate accelerometer measurements with UPDRS | 2018 |
| Kim et al. ^109^ | Rest tremor | 92 PD | SNUMAP, Life Science Technologies | Participants use wrist and finger sensor during rest position | Assess severity of symptoms using a convolutional neural network (CNN) on sensor readings | 2018 |
| Lipsmeier et al. ^133^ | Rest postural simple kinetic tremor | 44 PD 35 HS | Smartphone Galaxy S3 mini | For 6 months, participants completed tests eliciting tremor on smartphone once per day and carried smartphone passively during daily life | Assess the feasibility, reliability, and validity of smartphone-based digital biomarkers of PD in a clinical trial setting | 2018 |
| Zhou et al. ^251^ | Rest tremor | 18 PD | IMU: STEVAL-MKI108V2, STMicroelectronics | Participants perform activities that elicit tremor while using sensors | Characterize finger and wrist tremor in both the time and frequency domains and propose a tremor estimation algorithm | 2018 |
| Zhang et al. ^247^ | PD symptoms | 6 PD | - | Participants use accelerometer on wrist while performing ADLs | Automatically detect tremor in PD using accelerometers | 2018 |
| Heijmans et al. ^81^ | PD symptoms | 1 PD | IMU: MOX5 Maastricht Instruments | Participants use sensors during daytime | Predict tremor from wearable sensors in PD | 2019 |
| Zheng et al. ^249^ | Postural, simple kinetic, task specific tremor | 20 ET | Acc: Pebble smartwatch | Participants use smartwatch during activities that elicit tremor | Automaticaly detect ET severity using deep learning and smartwatches | 2019 |
| Loaiza Duque et al. ^136^ | Rest postural tremor | 16 ET 17 PD 12 HS 7 other | IPhone 5S | Participants use iPhone on hand while performing activities that elicit tremor | Use machine learning methods to analyze accelerometer readings to differentiate PD from ET | 2019 |
| Marino et al. ^148^ | Rest, Postural Tremor | 41 PD 36 HS | 4x 3-axis accelerometers ADXL345 | Participants wear 4 accelerometers, each placed on the finger tips while performing activities that elicit tremor | Quantify amplitude and frequency of PD tremor | 2019 |
| Hssayeni et al. ^91^ | PD upper limb impairment | 24 PD | Kinesia Sensors, Great Lakes Neurotech | Participants use a smarwatch while performing ADLs and UPDRS-III | Use machine learning algorithms to estimate tremor from smartwatch and correlate it to UPDRS-III | 2019 |
| Western et al. ^235^ | Intention tremor | 24 MS | IMU - Xbus Kit and MTw Kit, Xsens Technologies | Participants record FTN using IMU along their upper limbs | Correlate readings with FTMTRS | 2019 |
| Krishna et al. ^114^ | Intention tremor | 39 AT 31 HS | Biokin - MPU-9150 | Participants perform intention tremor activities such as FTN while using while using IMU on wrist | Principal component analysis of IMU readings | 2019 |
| López-Blanco et al. ^141^ | Rest Tremor | 22 PD | Sony Smartwatch 3 | Participants use smartwatches during tasks that elicit tremor | Correlate gyroscope readings with UPDRS-III | 2019 |
| Elm et al. ^56^ | PD symptoms | 51 PD | Smartwatch, Fox Wearable Companion App | Participants use smartwatch accelerometer paired with monitoring app during daily life | Create a dashboard for clinicians to classify different PD symptoms | 2019 |
| Isaacson et al. ^94^ | PD symptoms | 40 PD | Kinesia 360, Great Lakes Neurotech | Participants use Kinesia systems for 12 weeks and UPDRS is also evaluated | Continuous monitoring of PD symptoms after titrate medication | 2019 |
| Khodakarami et al. ^107^ | PD symptoms | 172 PD | Accelerometer: ADXL345 | Participants use wrist worn accelerometer during daily life | Distinguish which participants are suitable for device-assisted therapy based on accelerometer readings | 2019 |
| Musab et al. ^159^ | Rest, Postural Tremor | 10 PD | Shimmer 3 - IMU | Participants perform UPDRS-III while using IMU on wrist | Estimate PD symptoms using deep learning | 2019 |
| Tran et al. ^225^ | Intention tremor | 44 CA 14 HS | Kinect v2 | Participants use IMU and Kinect during a finger chase task and a FT task | Automatically evaluate motor impairment in CA | 2019 |
| Battista et al. ^14,15^ | PD symptoms | 20 PD | PD-Watch - Accelerometer | Participants use watch for 24 during daily life | Evaluate PD-Watch for continuous monitoring of PD symptoms | 2020 |
| Tran et al. ^224^ | Intention tremor | 7 AT 2 HS | Kinect and IMU: MPU9250 | Participants perform ballistic tracking task using IMU | Distinguish between AT and HS using motion capture | 2020 |
| Kashyap et al. ^103^ | Intention tremor | 23 AT 11 HS | Biokin - MPU-9150 and Kinect V2 | Participants perform different intention tremor activities such as FTN and FT while using IMU on wrist and positioned in front of Kinect | Principal component analysis of IMU and Kinect measurements in comparison with SARA score | 2020 |
| Mahadevan et al. ^146^ | Rest tremor, bradykinesia | 35 PD 60 HS | - | Participants use an accelerometer on the wrist while performing activities of UPDRS-III | Use machine learning in accelerometers to continuous monitor tremor and bradykinesia | 2020 |
| van Brummelen et al. ^27^ | Rest, Postural Tremor | 10 ET 10 PD | Smartwatch, smartphone, and Biometrics ACL300 3-ax accelerometer | Participants use a smartwatch/smartphone and a laboratory accelerometer while they perform activities that elicit tremor | Compare performance of consumer product and laboratory-grade accelerometers | 2020 |
| Erb et al. ^58^ | PD upper limb impairment | 95 PD 60 HS | IMU/EMG/ECG BioStamp RC, IMU GENEActiv | Participants use different sensors while performing part III of UPDRS-III and ADLs | Evaluate the use of wearables in clinical trials and as motor tracking in ADLs for PD | 2020 |
| Di Lazzaro et al. ^52^ | Rest, postural tremor | 36 PD 29 HS | IMU: Movit, Captiks Srl | Participants use sensors during activities that elicit tremor | Validate the use of IMUs to detect tremor and PD symptoms in recent diagnosed patients | 2020 |
| Shawen et al. ^205^ | Rest, Postural, Simple Kinetic, Task-specific, Intention tremor | 13 PD | BioStamp RC, Apple Watch Series 2 | Participants use wearable sensor and smartwatch on the same arm while performing activities that elicit tremor | Simplify wearable sensors by using machine learning methods | 2020 |
| Kavindya et al. ^104^ | Postural, task specific tremor | 15 PD/ET or Stroke | Soft glove with IMU MPU6050 | Participants use soft glove while performing activities that elicit tremor | Evaluate the use of a soft glove to quantify tremor | 2020 |
| Kwon et al. ^119^ | Postural tremor | 18 ET | Gyr: L3G4200D STMicroelectronics | Participants use gyroscope on upperlimb during stretch position | Quantify postural tremor in ET | 2020 |
| Locatelli et al. ^138^ | Rest, postural, simple kinetic, task specific tremor | 17 PD 7 ET | MuSe, acc: H3LIS331DL, gyr: ITG3701 | Participants perform activities that elicit tremor while using MuSe on hand | Validate MuSe to classify ET and PD | 2020 |
| Pahwa et al. ^171^ | PD symptoms | 26112 PD | KinetiGraph™ (PKG®) Movement Recording System | Anonymized data of a large PD population is analyzed | Evaluate the impact of using continuous objective measurement using the Personal KinetiGraph™ (PKG®) Movement Recording System in the routine clinical care of PD | 2020 |
| Sigcha et al. ^206^ | Rest tremor | 18 PD | Consumer smartwatch accelerometer | Participants use accelerometer during activities that elicit tremor | Evaluate amplitude and constancy of rest tremor using accelerometers | 2021 |
| Channa et al. ^35^ | Rest postural intention tremor | 20 PD 20 HS | IMU: Pmod NAV module | Participants use sensors during activities that elicit tremor | Objective assessment of tremor and bradykinesia and correlation to UPDRS | 2021 |
| Mcgurrin et al. ^150^ | Rest postural simple kinetic intention tremor | 13 ET | IMU: APDM Opal, USA | Participants use accelerometer and gyroscope on hand during TETRAS | Validate algorithm to score tremor in ET using inertial sensors | 2021 |
| Peres et al. ^182^ | Rest tremor | 15 PD 12 HS | from HS using IMUs | Participants use IMU on hand during rest position | Discrimination between early stages of PD | 2021 |
| Powers et al.^184^ | Rest tremor | 343 PD | Gyr and Acc : Apple Smartwatch | Participants use smartwatch long-term and measurements are correlated to MDS-UPDRS-III | Evaluate Motor fluctuations Monitor for Parkinson’s Disease (MM4PD), a system using smartwatch IMUs to continuously track rest tremor and dyskinesia | 2021 |
| Fuchs et al. ^67^ | Rest, Postural, Simple kinetic, Intention tremor | 20 ET | iPhone 5s accelerometer and gyroscope | Participants perform selected TETRAS tasks while they use smartphones strapped to their wrists | Quantify tremor severity and correlate it using fuzzy logic to TETRAS and QUEST tests | 2021 |
| Varghese et al. ^230^ | Postural, Rest, Simple Kinetic Tremor | 260 PD 89 HS 101 DD | Apple Smartwatch Series 3 and 4 | Participants use two smartwatches in each arm and perform tasks that elicit different types of tremor | Validate accelerometer and gyroscope sensors in smartwatch to predict PD vs HS and vs DD | 2021 |
| Teufl et al. ^219^ | Postural, Simple Kinetic, Task-specific tremor | 5 MS 10 HS | Accelerometer AX3 - Axivity | Subjects use a wrist-worn accelerometer while performing select FTMTRS and ARAT tasks | Detect MS tremor using FFT analysis | 2021 |
| Adam et al. ^1^ | Postural tremor | 24 MS 28 HS | MPU6050 - IMU | Participants use accelerometer on finger while stretching arms | Compare accelerometer parameters between HS and pwMS | 2021 |
| Hadley et al. ^78^ | PD symptoms | 16 PD | Kinesia sensors, CleveMed | Participants use for several days KinesiaU smartwatch | Assess clinical use of Kinesia sensors | 2021 |
| Kwon et al. ^121^ | Task specific tremor | 18 ET | Gyr: L3G4200D STMicroelectronics | Participants draw Archimedes spirals while using gyroscopes on upper limbs | Quantify task specific tremor during Archimedes spirals in ET | 2021 |
| Sun et al. ^214^ | PD symptoms | 30 PD | IMU MPU9250 | Participants use IMU on wrist while performing activities that elicit tremor | Evaluate the use of convolutional neural networks to distinguish rest, postural, and kinetic tremors | 2021 |
| Yuan et al. ^242^ | Rest tremor | 20 PD | - | Participants use accelerometer on wrist while performing activities that elicit tremor | Detect and quantify rest tremor in PD | 2021 |
| Channa et al. ^36^ | PD motor symptoms | 17 PD | Shimmer 3 - IMU | Participants perform UPDRS-III while using IMU on wrist | Estimate PD symptoms using deep learning | 2022 |
| Sahin et al. ^197^ | Postural tremor | 17 ET 9 PD 7 other (diabetes) | IMU: LPMS-B2 STD, Omni Instruments; App: Medoclinic | Participants use accelerometer during activities that elicit tremor | Characterize features of tremor in PD and ET using accelerometer and gyroscope | 2022 |
| Gauthier-Lafreniere et al. ^70^ | Rest postural tremor | 25 ET | GENEActiv Original -Activinsights | Participants use accelerometer during activities that elicit tremor | Develop a method to evaluate tremor using wrist-watch accelerometers in clinical exams | 2022 |
| Liu et al. ^135^ | Rest tremor | PD | 3 axial accelerometers | Participants use accelerometer during activities that elicit tremor | Quantify and monitor tremor severity compared to UPDRS | 2022 |
| Smid et al. ^209^ | Rest, postural, intention tremor | 28 PD 26 HS | Acc: MMA8452Q, Freescale Semiconductor | Participants use sensors at the index fingers while performing UPDRS | Translate UPDRS to an objective scoring using accelerometers | 2022 |
| Ricci et al. ^191^ | Rest, postural tremor | 36 PD | IMU: Moving G1, Captiks | Participants use sensors on whole body during activities that elicit tremor before, after 6 months and after 12 months of treatment | Objectify the impact of levodopa in PD motor symptoms | 2022 |
| Ali et al. ^4^ | Task specific tremor | 17 ET 18 HS | Acc: Delsys Trigno | Participants use sensors while drawing Archimedes spirals | Quantitative assessment of ET tremor and comparison with FTMRS | 2022 |
| Burq et al.^28^ | Rest tremor | 388 PD | Verily Study Watch, Verily Life Sciences | Participants use smartwatch during 390 days and perform activities eliciting PD symptoms twice a week | Validate the use of a smartwatch to remotely assess PD symptoms | 2022 |
| Ma et al. ^144,145^ | Postural tremor | 98 ET | IMU: MSP430 | Participants use IMU on hand during activities that elicit tremor | Quantify ET postural tremor severity | 2022 |
| Rabelo et al. ^189^ | Rest tremor | 17 PD | IMU: L3G4200D, LSM303DLM | Participants use accelerometer on hand in a rest position | Evaluate the use of accelerometers for low amplitude rest tremor | 2022 |
| Li et al.^127^ | Rest tremor | 16 PD | Acc: GeneActiv, Activinsights | Participants use wrist-worn accelerometer during activities that elicit tremor | Automatic label modification for tremor quantification in PD using machine learning methods | 2023 |
| Oyama et al. ^170^ | Rest, postural, simple kinetic tremor | 96 PD | Verily Study Watch, Verily Life Sciences | Participants use smartwatch during daily life and activities that elicit tremor | Validate continuous measurement of PD symptoms in population in Japan | 2023 |
| Kwon et al. ^120^ | Rest tremor | 9 SWEDD 11 PD | Gyro: L3G4200D, STMicroelectronics | Participants use gyroscope on fingers during activities that elicit tremor | Compare rest tremor in SWEDD versus PD | 2023 |
| Li et al. ^128^ | PD symptoms | 8 PD 30 HS | Pressure and flex sensors, IMU: MPU-9250 | Participants use sensory glove during different hand activities and postures | Detect tremor and hand kinematics in PD using a sensory glove | 2023 |
| Motion Capture Sensors - Electromagnetic Tracking and Force Transducters | | | | | | |
| O'Suilleabhain et al. ^169^ | Rest, Postural Tremor | 28 ET 23 PD 4 HS | 3Space Fastrak | Participants perform tasks eliciting postural and rest tremor tasks with electromagnetic tracking system | Evaluate the correlation between measured tremor amplitude and clinician visual estimate and its accuracy with simulated tremor | 2001 |
| Rozman et al. ^196^ | Rest tremor | 8 PD | Wheatstone bridge - 1-LY41-10/700 force transducter | Participants use force transducters on wrist and fingers during rest activities | Develop a quantitative method to measure tremor amplitude using force sensors | 2007 |
| Pradhan et al. ^185^ | PD Symptoms | 30 PD 30 HS | Force sensor Nano17; ATI Automation Industries, Apex | Participants use force transducters on fingers during pinch and grasp | Develop a quantitative method to measure tremor amplitude using force sensors | 2010 |
| Patel et al. ^179^ | Postural, simple kinetic, task specific tremor | 5PD | Electromagnetic motion tracker: Liberty Polhemus, angle tracker: cyberglovem, emg: Delsys Trigno | Participants use multisnsory system during activities that elicit tremor | Evaluate multisensory system to track PD motor symptoms | 2016 |
| Charles et al. ^37^ | Postural Tremor | 10 ET | trakSTAR - Ascension Technologies | Motion capture in 16 different upper limb postures using electromagnetic sensors and EMG | Create a computational model of upper limb tremor propagation in ET | 2017 |
| Van den Noort et al. ^166^ | PD symptoms | 4 PD | Acc, Gyr: LSM330DLC; Force: ATI mini45 | Participants used powerglove system while performing activities that elicit tremor | Quantify PD symptoms using IMUs and force sensors | 2017 |
| Perera et al. ^181^ | Rest postural simple kinetic intention tremor | 13 MS 12 HS | TREMBAL electromagnetic sensors | Participants use motion tracker during activities that elicit tremor | Correlate electromagnetic motion capture with clinical tests and minimum detectable changes | 2019 |
| Dai et al. ^44^ | Rest, Postural, Simple kinetic tremor | 45 PD 30 HS | EMTS, NDI Aurora | Participants use a 6 axis electromagnetic tracking device on the index finger tip while performing activities that elicit tremor | Quantify tremor and bradykinesia and correlate it with UPDRS | 2021 |
| Motion Capture Sensors - Camera based | | | | | | |
| Deuschl et al. ^51^ | Intention Tremor | 26 ET 9 MS 3 AT 12 HS | MacReflex version 3.2, Qualisys | Participants grasp a target with their thumb and index at 34 cm above their rest position | Observe intention tremor in ET and compare it to people with cerebellar disease | 2000 |
| Das et al. ^47^ | Rest intention tremor | 4 PD 2 HS | Vicon infrared cameras | Participants use motion capture system while performing motor activities | Quantify PD motor symptoms through motion capture | 2011 |
| Chen et al. ^38^ | Postural Tremor | 4 ET | Leap Motion Device | Participants sit on a chair with one arm stretched such that fingers are 12-16 cm above the device | Quantify tremors using measured amplitude and frequency of finger movement | 2016 |
| Casacanditella et al. ^33^ | Rest, postural tremor | 2 PD 1 ET | Kinect and Laser Doppler Vibrometer PDV 100 | Participants sit in front of Kinect and vibrometer while doing activities that elicit tremor | Assess the feasibility of using a Kinect and a vibrometer to measure tremor | 2017 |
| Lugo et al. ^142^ | Postural, simple kinetic tremor | 33 PD | Leap Motion Device | Participants perform different virtual reality games controlled by Leap Motion Sensor | Validate the use of virtual reality and Leap Motion systems to evaluate tremor | 2017 |
| Li et al. ^126^ | PD symptoms | 9 PD | Camera recording processed for markerless pose estimation | Participants perform selected UPDRS activities in front of a video camera | Evaluate the feasibility of vision-based assessment of PD using pose estimation | 2018 |
| Saraguro et al. ^201^ | Simple kinetic tremor | 4 PD 2 HS | Kinect v2 | Participants perform FT in front of kinect | Evaluate the use of FT with Kinect to assess tremor | 2019 |
| Mitsui et al. ^154^ | Intention tremor | 10 ET 10 AT | Smartphone camera - computer vision | Participants perform FTN in front of smartphone camera | Automatically and objectively differentiate ET from cerebellar disease | 2020 |
| Pang et al. ^174^ | Rest, postural, simple kinetic tremor | 5 PD 22 HS | OpenPose, 2 Logitech HD Pro C920 webcams (1080p) | Participants perform hand movements in front of two web cameras in a closed box with white background | Markerless pose estimation of hand movements using discrete wavelet decomposition (DWT) to identify tremor and bradykinesia in PD | 2020 |
| Kim et al. ^111^ | Rest tremor | 2 PD | Leap Motion Device | Participants sit on a chair with one hand above the device | Quantify tremors using measured amplitude and frequency of hand movement | 2020 |
| Williams et al.^238^ | Rest, postural tremor | 9 PD 5 ET 1 Other | Video: Smartphone, Acc: Natus Neurology Tremor Sensor | Participants use accelerometer on hand and videos are recorded during activities that elicit tremor | Compare computer vision techniques with accelerometer for tremor detection | 2020 |
| Khwaounjoo et al. ^108^ | Postural tremor | 55 PD | Leap Motion Device | Participants extend hands on top of leap motion sensor | Investigate which location on the hand maximizes tremor severity | 2022 |
| Park et al. ^177^ | PD Symptoms | 18 HS | Zoom – MediaPipe | Participants perform FT during Zoom videocall | Evaluate the feasibility of markerless hand pose estimation to assess movement in videocalls | 2023 |
| Ismail et al. ^95^ | Postural tremor | 12 ET | Smartphone camera and Instagram App | Participants use instagram filter where they keep their hand in a steady, against gravity position | Correlation between "steady-hand" instagram filter and Archimedes spirals | 2023 |

*Abbreviations:* RSD: Reﬂex Sympathetic Dystrophy; ET, Essential Tremor; PD, Parkinson’s Disease; MS, Multiple Sclerosis; AT, Ataxia; HS, Healthy Subject; HD, Huntington’s Disease; PH, Physiological tremor; ART: Age-related tremor; MSA-C, Multiple system atrophy cerebellar subtype; DD: Diﬀerential Diagnosis (used when speciﬁc condition is not mentioned, e.g., for^230^ mixed group with MS, AT and ET; for^68^ mixed group with ET, PD, and cerebellar disease); SWEDD: Scans Without Evidence of Dopaminergic Deﬁcits; CA: Cerebellar Ataxia

EMG, Superﬁcial Electromyography; MMG, Mechanomyography; IMU, Inertial Measurement Unit; EEG, Electroencephalogram; TMS, Transcranial Magnetic Stimulation; ECG, Electrocardiogram;

FTMTRS, Fahn-Tolosa-Marin Tremor Rating; UPDRS-III, Movement Disorder Society Uniﬁed Parkinson’s Disease Rating Scale - Part III; TETRAS, Essential Tremor Rating Assessment Scale; FT, Finger Tapping; DaS, Draw a Shape test; SARA, Scale for the Assessment and Rating of Ataxia; ADLs, Activities of Daily Living; ARAT, Action Research Arm Test; QUEST, quality of life in ET questionnaire

VPIT, virtual peg insertion test; CNN, convolutional neural networks;

FFT, fast Fourier transform; PSD, power spectral density; SVM, support vector machine; ML, machine learning; ERP, event-related potentials; ERD, event-related desynchronization; ERS, event-related synchronization; EMD, empirical mode decomposition; WFLC, weighted-frequency Fourier linear combiner; KF, Kalman filter; EKF, extended Kalman filter; HHT, Hilbert-Huang transform; DOF, degree of freedom; BMFLC: band-limited multiple Fourier linear combiner

1. Adam, V., and J. Havlík. Parameterization of the Tremor Signal from Accelerometers in Multiple Sclerosis. , 2021.doi:10.23919/AE51540.2021.9542904

2. Aghanavesi, S., D. Nyholm, M. Senek, F. Bergquist, and M. Memedi. A smartphone-based system to quantify dexterity in Parkinson’s disease patients. *Informatics in Medicine Unlocked* 9:11–17, 2017.

3. Aisen, M. L., and N. G. La Rocca. Quantitative Assessment of Tremor in Multiple Sclerosis Patients: A New Technique. *Assistive Technology* 1:3–6, 1989.

4. Ali, S. M., S. P. Arjunan, J. Peters, L. Perju-Dumbrava, C. Ding, M. Eller, S. Raghav, P. Kempster, M. A. Motin, P. J. Radcliffe, and D. K. Kumar. Wearable sensors during drawing tasks to measure the severity of essential tremor. *Sci Rep* 12:5242, 2022.

5. Angeles, P., Y. Tai, N. Pavese, S. Wilson, and R. Vaidyanathan. Automated assessment of symptom severity changes during deep brain stimulation (DBS) therapy for Parkinson’s disease. , 2017.doi:10.1109/ICORR.2017.8009462

6. Aoh, Y., H.-J. Hsiao, M.-K. Lu, A. Macerollo, H.-C. Huang, M. Hamada, C.-H. Tsai, and J.-C. Chen. Event-Related Desynchronization/Synchronization in Spinocerebellar Ataxia Type 3. *Frontiers in Neurology* 10:, 2019.

7. Atashzar, S. F., M. Shahbazi, O. Samotus, M. Tavakoli, M. S. Jog, and R. V. Patel. Characterization of Upper-Limb Pathological Tremors: Application to Design of an Augmented Haptic Rehabilitation System. *IEEE Journal of Selected Topics in Signal Processing* 10:888–903, 2016.

8. Ayache, S. S., T. Al-ani, and J.-P. Lefaucheur. Distinction between essential and physiological tremor using Hilbert-Huang transform. *Neurophysiologie Clinique/Clinical Neurophysiology* 44:203–212, 2014.

9. Ayache, S. S., M. A. Chalah, T. Al-Ani, W. H. Farhat, H. G. Zouari, A. Créange, and J.-P. Lefaucheur. Tremor in multiple sclerosis: The intriguing role of the cerebellum. *Journal of the Neurological Sciences* 358:351–356, 2015.

10. Bacher, M., E. Scholz, and H. C. Diener. 24 Hour continuous tremor quantification based on EMG recording. *Electroencephalography and Clinical Neurophysiology* 72:176–183, 1989.

11. Bai, Q., T. Shen, B. Xu, Q. Yu, H. Zhang, C. Mao, C. Liu, and S. Wang. Quantification of the motor symptoms of Parkinson’s disease. , 2017.doi:10.1109/NER.2017.8008297

12. Barrantes, S., A. J. S. Egea, H. A. G. Rojas, M. J. Martí, Y. Compta, F. Valldeoriola, E. S. Mezquita, E. Tolosa, and J. Valls-Solè. Differential diagnosis between Parkinson’s disease and essential tremor using the smartphone’s accelerometer. *PLOS ONE* 12:e0183843, 2017.

13. Basu, I., D. Graupe, D. Tuninetti, P. Shukla, K. V. Slavin, L. V. Metman, and D. M. Corcos. Pathological tremor prediction using surface electromyogram and acceleration: potential use in ‘ON–OFF’ demand driven deep brain stimulator design. *J. Neural Eng.* 10:036019, 2013.

14. Battista, L., and A. Romaniello. A novel device for continuous monitoring of tremor and other motor symptoms. *Neurol Sci* 39:1333–1343, 2018.

15. Battista, L., and A. Romaniello. A wearable tool for selective and continuous monitoring of tremor and dyskinesia in Parkinsonian patients. *Parkinsonism & Related Disorders* 77:43–47, 2020.

16. Bazgir, O., J. Frounchi, S. A. H. Habibi, L. Palma, and P. Pierleoni. A neural network system for diagnosis and assessment of tremor in parkinson disease patients. , 2015.doi:10.1109/ICBME.2015.7404105

17. Bazgir, O., S. A. H. Habibi, L. Palma, P. Pierleoni, and S. Nafees. A Classification System for Assessment and Home Monitoring of Tremor in Patients with Parkinson’s Disease. *J Med Signals Sens* 8:65–72, 2018.

18. Bermeo, A., M. Bravo, M. Huerta, and A. Soto. A system to monitor tremors in patients with Parkinson’s disease. , 2016.doi:10.1109/EMBC.2016.7591852

19. Beuter, A., A. De Geoffroy, and P. Cordo. The measurement of tremor using simple laser systems. *Journal of Neuroscience Methods* 53:47–54, 1994.

20. Bhidayasiri, R., S. Petchrutchatachart, R. Pongthornseri, C. Anan, S. Dumnin, and C. Thanawattano. Low-Cost, 3-Dimension, Office-Based Inertial Sensors for Automated Tremor Assessment: Technical Development and Experimental Verification. *Journal of Parkinson’s Disease* 4:273–282, 2014.

21. Boroojerdi, B., R. Ghaffari, N. Mahadevan, M. Markowitz, K. Melton, B. Morey, C. Otoul, S. Patel, J. Phillips, E. Sen-Gupta, O. Stumpp, D. Tatla, D. Terricabras, K. Claes, J. A. Wright, and N. Sheth. Clinical feasibility of a wearable, conformable sensor patch to monitor motor symptoms in Parkinson’s disease. *Parkinsonism & Related Disorders* 61:70–76, 2019.

22. Bosch, T. J., C. Groth, and A. Singh. Resting-State Low-Frequency Cerebellar Oscillations Can Be Abnormal in Parkinson’s Disease. *Cerebellum* 21:1139–1143, 2022.

23. Boukhvalova, A. K., O. Fan, A. M. Weideman, T. Harris, E. Kowalczyk, L. Pham, P. Kosa, and B. Bielekova. Smartphone Level Test Measures Disability in Several Neurological Domains for Patients With Multiple Sclerosis. *Frontiers in Neurology* 10:, 2019.

24. Bravo, M., A. Bermeo, M. Huerta, C. Llumiguano, J. Bermeo, R. Clotet, and A. Soto. A system for finger tremor quantification in patients with Parkinson’s disease. , 2017.doi:10.1109/EMBC.2017.8037623

25. Breit, S., S. Spieker, J. B. Schulz, and T. Gasser. Long-term EMG recordings differentiate between parkinsonian and essential tremor. *J Neurol* 255:103–111, 2008.

26. Brennan, K. C., E. C. Jurewicz, B. Ford, S. L. Pullman, and E. D. Louis. Is essential tremor predominantly a kinetic or a postural tremor? A clinical and electrophysiological study. *Movement Disorders* 17:313–316, 2002.

27. van Brummelen, E. M. J., D. Ziagkos, W. M. I. de Boon, E. P. Hart, R. J. Doll, T. Huttunen, P. Kolehmainen, and G. J. Groeneveld. Quantification of tremor using consumer product accelerometry is feasible in patients with essential tremor and Parkinson’s disease: a comparative study. *Journal of Clinical Movement Disorders* 7:4, 2020.

28. Burq, M., E. Rainaldi, K. C. Ho, C. Chen, B. R. Bloem, L. J. W. Evers, R. C. Helmich, L. Myers, W. J. Marks, and R. Kapur. Virtual exam for Parkinson’s disease enables frequent and reliable remote measurements of motor function. *npj Digit. Med.* 5:65, 2022.

29. Cai, G., Z. Lin, H. Dai, X. Xia, Y. Xiong, S.-J. Horng, and T. C. Lueth. Quantitative assessment of parkinsonian tremor based on a linear acceleration extraction algorithm. *Biomedical Signal Processing and Control* 42:53–62, 2018.

30. Caligiuri, M., and R. Tripp. A portable hand-held device for quantifying and standardizing tremor assessment. *Journal of Medical Engineering & Technology* 28:254–262, 2004.

31. Carpinella, I., D. Cattaneo, and M. Ferrarin. Quantitative assessment of upper limb motor function in Multiple Sclerosis using an instrumented Action Research Arm Test. *Journal of NeuroEngineering and Rehabilitation* 11:67, 2014.

32. Carpinella, I., D. Cattaneo, and M. Ferrarin. Hilbert–Huang transform based instrumental assessment of intention tremor in multiple sclerosis. *J. Neural Eng.* 12:046011, 2015.

33. Casacanditella, L., G. Cosoli, M. G. Ceravolo, and E. P. Tomasini. Non-contact measurement of tremor for the characterisation of Parkinsonian individuals: comparison between Kinect and Laser Doppler vibrometer. *J. Phys.: Conf. Ser.* 882:012002, 2017.

34. Chan, P. Y., Z. M. Ripin, S. A. Halim, J. Tharakan, M. Muzaimi, K. S. Ng, M. I. Kamarudin, G. B. Eow, J. Y. Hor, K. Tan, C. F. Cheah, N. Soong, L. Then, and A. S. Yahya. An In–Laboratory Validity and Reliability Tested System for Quantifying Hand–Arm Tremor in Motions. *IEEE Trans. Neural Syst. Rehabil. Eng.* 26:460–467, 2018.

35. Channa, A., R.-C. Ifrim, D. Popescu, and N. Popescu. A-WEAR Bracelet for Detection of Hand Tremor and Bradykinesia in Parkinson’s Patients. *Sensors* 21:981, 2021.

36. Channa, A., G. Ruggeri, N. Mammone, R.-C. Ifrim, A. Iera, and N. Popescu. Parkinson’s Disease Severity Estimation using Deep Learning and Cloud Technology. , 2022.doi:10.1109/COINS54846.2022.9854945

37. Charles, S. K., D. W. Geiger, A. D. Davidson, A. C. Pigg, C. P. Curtis, and B. C. Allen. Toward quantitative characterization of essential tremor for future tremor suppression. , 2017.doi:10.1109/ICORR.2017.8009242

38. Chen, K.-H., P.-C. Lin, Y.-J. Chen, B.-S. Yang, and C.-H. Lin. Development of method for quantifying essential tremor using a small optical device. *Journal of Neuroscience Methods* 266:78–83, 2016.

39. Cleeves, L., and L. J. Findley. Variability in amplitude of untreated essential tremor. *Journal of Neurology, Neurosurgery & Psychiatry* 50:704–708, 1987.

40. Cohen, O., S. Pullman, E. Jurewicz, D. Watner, and E. D. Louis. Rest Tremor in Patients With Essential Tremor: Prevalence, Clinical Correlates, and Electrophysiologic Characteristics. *Archives of Neurology* 60:405–410, 2003.

41. Cole, B. T., S. H. Roy, C. J. De Luca, and S. H. Nawab. Dynamical Learning and Tracking of Tremor and Dyskinesia From Wearable Sensors. *IEEE Transactions on Neural Systems and Rehabilitation Engineering* 22:982–991, 2014.

42. Contreras, R., M. Huerta, G. Sagbay, C. LLumiguano, M. Bravo, A. Bermeo, R. Clotet, and A. Soto. Tremors quantification in parkinson patients using smartwatches. , 2016.doi:10.1109/ETCM.2016.7750866

43. Creagh, A. P., C. Simillion, A. Scotland, F. Lipsmeier, C. Bernasconi, S. Belachew, J. van Beek, M. Baker, C. Gossens, M. Lindemann, and M. D. Vos. Smartphone-based remote assessment of upper extremity function for multiple sclerosis using the Draw a Shape Test. *Physiol. Meas.* 41:054002, 2020.

44. Dai, H., G. Cai, Z. Lin, Z. Wang, and Q. Ye. Validation of Inertial Sensing-Based Wearable Device for Tremor and Bradykinesia Quantification. *IEEE Journal of Biomedical and Health Informatics* 25:997–1005, 2021.

45. Dai, H., P. Zhang, and T. C. Lueth. Quantitative Assessment of Parkinsonian Tremor Based on an Inertial Measurement Unit. *Sensors* 15:25055–25071, 2015.

46. Daneault, J.-F., B. Carignan, C. É. Codère, A. Sadikot, and C. Duval. Using a Smart Phone as a Standalone Platform for Detection and Monitoring of Pathological Tremors. *Frontiers in Human Neuroscience* 6:, 2013.

47. Das, S., L. Trutoiu, A. Murai, D. Alcindor, M. Oh, F. De la Torre, and J. Hodgins. Quantitative measurement of motor symptoms in Parkinson’s disease: A study with full-body motion capture data. , 2011.doi:10.1109/IEMBS.2011.6091674

48. DelMastro, H. M., J. A. Ruiz, E. S. Gromisch, J. C. Garbalosa, E. W. Triche, K. M. Olson, and A. C. Lo. Quantification characteristics of digital spiral analysis for understanding the relationship among tremor and clinical measures in persons with multiple sclerosis. *Journal of Neuroscience Methods* 307:254–259, 2018.

49. Delrobaei, M., S. Memar, M. Pieterman, T. W. Stratton, K. McIsaac, and M. Jog. Towards remote monitoring of Parkinson’s disease tremor using wearable motion capture systems. *Journal of the Neurological Sciences* 384:38–45, 2018.

50. Deuschl, G., H. Blumberg, and C. H. Lücking. Tremor in Reflex Sympathetic Dystrophy. *Archives of Neurology* 48:1247–1252, 1991.

51. Deuschl, G., R. Wenzelburger, K. Löffler, J. Raethjen, and H. Stolze. Essential tremor and cerebellar dysfunction Clinical and kinematic analysis of intention tremor. *Brain* 123:1568–1580, 2000.

52. Di Lazzaro, G., M. Ricci, M. Al-Wardat, T. Schirinzi, S. Scalise, F. Giannini, N. B. Mercuri, G. Saggio, and A. Pisani. Technology-Based Objective Measures Detect Subclinical Axial Signs in Untreated, de novo Parkinson’s Disease. *JPD* 10:113–122, 2020.

53. Elble, R. J., M. Brilliant, K. Leffler, and C. Higgins. Quantification of essential tremor in writing and drawing. *Movement Disorders* 11:70–78, 1996.

54. Elble, R. J., and A. Ellenbogen. Digitizing Tablet and Fahn–Tolosa–Marín Ratings of Archimedes Spirals have Comparable Minimum Detectable Change in Essential Tremor. 7:481, 2017.

55. Elble, R. J., R. Sinha, and C. Higgins. Quantification of tremor with a digitizing tablet. *Journal of Neuroscience Methods* 32:193–198, 1990.

56. Elm, J. J., M. Daeschler, L. Bataille, R. Schneider, A. Amara, A. J. Espay, M. Afek, C. Admati, A. Teklehaimanot, and T. Simuni. Feasibility and utility of a clinician dashboard from wearable and mobile application Parkinson’s disease data. *npj Digit. Med.* 2:1–6, 2019.

57. Erasmus, L.-P., S. Sarno, H. Albrecht, M. Schwecht, W. Pöllmann, and N. König. Measurement of ataxic symptoms with a graphic tablet: standard values in controls and validity in Multiple Sclerosis patients. *Journal of Neuroscience Methods* 108:25–37, 2001.

58. Erb, M. K., D. R. Karlin, B. K. Ho, K. C. Thomas, F. Parisi, G. P. Vergara-Diaz, J.-F. Daneault, P. W. Wacnik, H. Zhang, T. Kangarloo, C. Demanuele, C. R. Brooks, C. N. Detheridge, N. Shaafi Kabiri, J. S. Bhangu, and P. Bonato. mHealth and wearable technology should replace motor diaries to track motor fluctuations in Parkinson’s disease. *npj Digit. Med.* 3:1–10, 2020.

59. Ferenčík, N., M. Jaščur, M. Bundzel, and F. Cavallo. The Rehapiano—Detecting, Measuring, and Analyzing Action Tremor Using Strain Gauges. *Sensors* 20:663, 2020.

60. Ferleger, B. I., K. S. Sonnet, T. H. Morriss, A. L. Ko, H. J. Chizeck, and J. A. Herron. A tablet- and mobile-based application for remote diagnosis and analysis of movement disorder symptoms. , 2020.doi:10.1109/EMBC44109.2020.9176044

61. Ferreira, J. J., C. Godinho, A. T. Santos, J. Domingos, D. Abreu, R. Lobo, N. Gonçalves, M. Barra, F. Larsen, Ø. Fagerbakke, I. Akeren, H. Wangen, J. A. Serrano, P. Weber, A. Thoms, S. Meckler, S. Sollinger, J. van Uem, M. A. Hobert, K. S. Maier, H. Matthew, T. Isaacs, J. Duffen, H. Graessner, and W. Maetzler. Quantitative home-based assessment of Parkinson’s symptoms: The SENSE-PARK feasibility and usability study. *BMC Neurology* 15:89, 2015.

62. Feys, P., W. F. Helsen, A. Lavrysen, B. Nuttin, and P. Ketelaer. Intention tremor during manual aiming: a study of eye and hand movements. *Multiple Sclerosis* 11.

63. Feys, P., W. F. Helsen, X. Liu, A. Lavrysen, V. Loontjens, B. Nuttin, and P. Ketelaer. Effect of visual information on step-tracking movements in patients with intention tremor due to multiple sclerosis. *Mult Scler* 9:492–502, 2003.

64. Feys, P., W. Helsen, A. Prinsmel, S. Ilsbroukx, S. Wang, and X. Liu. Digitised spirography as an evaluation tool for intention tremor in multiple sclerosis. *Journal of Neuroscience Methods* 160:309–316, 2007.

65. Foerster, F., and M. Smeja. Joint amplitude and frequency analysis of tremor activity. *Electromyogr Clin Neurophysiol* 39:11–19, 1999.

66. Fraiwan, L., R. Khnouf, and A. R. Mashagbeh. Parkinson’s disease hand tremor detection system for mobile application. *Journal of Medical Engineering & Technology* 40:127–134, 2016.

67. Fuchs, C., M. S. Nobile, G. Zamora, A. Degeneffe, P. Kubben, and U. Kaymak. Tremor assessment using smartphone sensor data and fuzzy reasoning. *BMC Bioinformatics* 22:57, 2021.

68. Gallego, J. A., E. Rocon, J. Ibañez, J. L. Dideriksen, A. D. Koutsou, R. Paradiso, M. B. Popovic, J. M. Belda-Lois, F. Gianfelici, D. Farina, D. B. Popovic, M. Manto, T. D’Alessio, and J. L. Pons. A soft wearable robot for tremor assessment and suppression. , 2011.doi:10.1109/ICRA.2011.5979639

69. Gallego, J. A., E. Rocon, J. O. Roa, J. C. Moreno, and J. L. Pons. Real-Time Estimation of Pathological Tremor Parameters from Gyroscope Data. *Sensors* 10:2129–2149, 2010.

70. Gauthier-Lafreniere, E., M. Aljassar, V. V. Rymar, J. Milton, and A. F. Sadikot. A standardized accelerometry method for characterizing tremor: Application and validation in an ageing population with postural and action tremor. *Front. Neuroinform.* 16:878279, 2022.

71. Ghassemi, N. H., F. Marxreiter, C. F. Pasluosta, P. Kugler, J. Schlachetzki, A. Schramm, B. M. Eskofier, and J. Klucken. Combined accelerometer and EMG analysis to differentiate essential tremor from Parkinson’s disease. , 2016.doi:10.1109/EMBC.2016.7590791

72. Giuffrida, J. P., D. E. Riley, B. N. Maddux, and D. A. Heldman. Clinically deployable Kinesia^TM^ technology for automated tremor assessment. *Movement Disorders* 24:723–730, 2009.

73. Goetz, C. G., G. T. Stebbins, D. Wolff, W. DeLeeuw, H. Bronte-Stewart, R. Elble, M. Hallett, J. Nutt, L. Ramig, T. Sanger, A. D. Wu, P. H. Kraus, L. M. Blasucci, E. A. Shamim, K. D. Sethi, J. Spielman, K. Kubota, A. S. Grove, E. Dishman, and C. B. Taylor. Testing objective measures of motor impairment in early Parkinson’s disease: Feasibility study of an at-home testing device. *Mov Disord.* 24:551–556, 2009.

74. Gorbunov, A., Y. Gromov, E. Dolgov, E. Tugolukov, and A. Neprokin. Accelerometric Studies of Night-Time Motor Activity with Essential Tremor. , 2020.doi:10.1109/SUMMA50634.2020.9280755

75. Graves, J. S., M. Ganzetti, F. Dondelinger, F. Lipsmeier, S. Belachew, C. Bernasconi, X. Montalban, J. van Beek, M. Baker, C. Gossens, and M. Lindemann. Preliminary validity of the Draw a Shape Test for upper extremity assessment in multiple sclerosis. *Annals of Clinical and Translational Neurology* n/a:

76. Gulde, P., M. Cetin, J. Hermsdörfer, and P. Rieckmann. Changes in thumb tapping rates and central motor conduction times are associated in persons with multiple sclerosis. *Neurol Sci* 43:4945–4951, 2022.

77. Hacisalihzade, S. S., C. Albani, and M. Mansour. Measuring parkinsonian symptoms with a tracking device. *Computer Methods and Programs in Biomedicine* 27:257–268, 1988.

78. Hadley, A. J., D. E. Riley, and D. A. Heldman. Real-World Evidence for a Smartwatch-Based Parkinson’s Motor Assessment App for Patients Undergoing Therapy Changes. *Digital Biomarkers* 5:206–215, 2021.

79. Haubenberger, D., D. Kalowitz, F. B. Nahab, C. Toro, D. Ippolito, D. A. Luckenbaugh, L. Wittevrongel, and M. Hallett. Validation of digital spiral analysis as outcome parameter for clinical trials in essential tremor. *Movement Disorders* 26:2073–2080, 2011.

80. Havlík, J., T. Szentpétery, M. Němečková, D. Vávrová, K. Řasová, J. Zeman, and P. Sovka. Design and realization of measuring device for tremor evaluation. , 2015.doi:10.1109/IWCIM.2015.7347079

81. Heijmans, M., J. Habets, M. Kuijf, P. Kubben, and C. Herff. Evaluation of Parkinson’s Disease at Home: Predicting Tremor from Wearable Sensors. , 2019.doi:10.1109/EMBC.2019.8857717

82. Heldman, D. A., A. J. Espay, P. A. LeWitt, and J. P. Giuffrida. Clinician versus machine: Reliability and responsiveness of motor endpoints in Parkinson’s disease. *Parkinsonism & Related Disorders* 20:590–595, 2014.

83. Heldman, D. A., D. A. Harris, T. Felong, K. L. Andrzejewski, E. R. Dorsey, J. P. Giuffrida, B. Goldberg, and M. A. Burack. Telehealth Management of Parkinson’s Disease Using Wearable Sensors: An Exploratory Study. *Digit Biomark* 1:43–51, 2017.

84. Heldman, D. A., J. Jankovic, D. E. Vaillancourt, J. Prodoehl, R. J. Elble, and J. P. Giuffrida. Essential tremor quantification during activities of daily living. *Parkinsonism & Related Disorders* 17:537–542, 2011.

85. Hoff, J. I., V. van der Meer, and J. J. van Hilten. Accuracy of objective ambulatory accelerometry in detecting motor complications in patients with Parkinson disease. *Clin Neuropharmacol* 27:53–57, 2004.

86. Hossen, A., Z. Al-Hakim, M. Muthuraman, J. Raethjen, G. Deuschl, and U. Heute. Discrimination of Parkinsonian Tremor From Essential Tremor by Voting Between Different EMG Signal Processing Techniques. *Jou. Eng. Res.* 11:11–22, 2014.

87. Hossen, A., A. R. Anwar, N. Koirala, H. Ding, D. Budker, A. Wickenbrock, U. Heute, G. Deuschl, S. Groppa, and M. Muthuraman. Machine learning aided classification of tremor in multiple sclerosis. *eBioMedicine* 82:104152, 2022.

88. Hossen, A., G. Deuschl, S. Groppa, U. Heute, and M. Muthuraman. Discrimination of physiological tremor from pathological tremor using accelerometer and surface EMG signals. *Technology and Health Care* 28:461–476, 2020.

89. Hossen, A., M. Muthuraman, Z. Al-Hakim, J. Raethjen, G. Deuschl, and U. Heute. Discrimination of Parkinsonian tremor from essential tremor using statistical signal characterization of the spectrum of accelerometer signal. *Bio-Medical Materials and Engineering* 23:513–531, 2013.

90. Hossen, A., M. Muthuraman, J. Raethjen, G. Deuschl, and U. Heute. Discrimination of Parkinsonian tremor from essential tremor by implementation of a wavelet-based soft-decision technique on EMG and accelerometer signals. *Biomedical Signal Processing and Control* 5:181–188, 2010.

91. Hssayeni, M. D., J. Jimenez-Shahed, M. A. Burack, and B. Ghoraani. Wearable Sensors for Estimation of Parkinsonian Tremor Severity during Free Body Movements. *Sensors* 19:4215, 2019.

92. Huo, W., P. Angeles, Y. F. Tai, N. Pavese, S. Wilson, M. T. Hu, and R. Vaidyanathan. A Heterogeneous Sensing Suite for Multisymptom Quantification of Parkinson’s Disease. *IEEE Transactions on Neural Systems and Rehabilitation Engineering* 28:1397–1406, 2020.

93. Ibáñez, J., J. I. Serrano, M. D. Del Castillo, J. A. Gallego, and E. Rocon. Online detector of movement intention based on EEG—Application in tremor patients. *Biomedical Signal Processing and Control* 8:822–829, 2013.

94. Isaacson, S. H. *et al.* Prospective Home-use Study on Non-invasive Neuromodulation Therapy for Essential Tremor. *Tremor and Other Hyperkinetic Movements* 10:29, 2020.

95. Ismail, I. I., W. A. Kamel, and J. Y. Al‐Hashel. Assessing the Usability of an Instagram Filter in Monitoring Essential Tremor: A Proof‐of‐Concept Study. *Movement Disord Clin Pract* 10:274–278, 2023.

96. Iwasaki, Y., T. Hirotomi, H. Oguro, and M. Nakamura. Preliminary Study on Using Accelerometers to Measure Involuntary Movements for the Assessment of Neurological Motor Impairments. , 2013.doi:10.1109/IIAI-AAI.2013.46

97. Jankovic, J., K. S. Schwartz, and W. Ondo. Re-emergent tremor of Parkinson’s disease. *Journal of Neurology, Neurosurgery & Psychiatry* 67:646–650, 1999.

98. Jeon, H., W. Lee, H. Park, H. J. Lee, S. K. Kim, H. B. Kim, B. Jeon, and K. S. Park. Automatic Classification of Tremor Severity in Parkinson’s Disease Using a Wearable Device. *Sensors* 17:2067, 2017.

99. Joundi, R. A., J.-S. Brittain, N. Jenkinson, A. L. Green, and T. Aziz. Rapid tremor frequency assessment with the iPhone accelerometer. *Parkinsonism & Related Disorders* 17:288–290, 2011.

100. Júnior, E. P., I. L. D. Delmiro, N. Magaia, F. M. Maia, M. M. Hassan, V. H. C. Albuquerque, and G. Fortino. Intelligent Sensory Pen for Aiding in the Diagnosis of Parkinson’s Disease from Dynamic Handwriting Analysis. *Sensors* 20:5840, 2020.

101. Kanzler, C. M., I. Lamers, P. Feys, R. Gassert, and O. Lambercy. Personalized prediction of rehabilitation outcomes in multiple sclerosis: a proof-of-concept using clinical data, digital health metrics, and machine learning. *Med Biol Eng Comput* 60:249–261, 2022.

102. Kanzler, C. M., I. Lessard, R. Gassert, B. Brais, C. Gagnon, and O. Lambercy. Reliability and validity of digital health metrics for assessing arm and hand impairments in an ataxic disorder. *Ann Clin Transl Neurol* 9:432–443, 2022.

103. Kashyap, B., D. Phan, P. N. Pathirana, M. Horne, L. Power, and D. Szmulewicz. Objective Assessment of Cerebellar Ataxia: A Comprehensive and Refined Approach. *Sci Rep* 10:9493, 2020.

104. Kavindya, P., W. V. I. Awantha, A. T. Wanasinghe, A. L. Kulasekera, D. S. Chathuranga, and B. Senanayake. Evaluation of Hand Tremor Frequency Among Patients in Sri Lanka using a Soft Glove. , 2020.doi:10.1109/MERCon50084.2020.9185382

105. Keijsers, N. L. W., M. W. I. M. Horstink, and S. C. A. M. Gielen. Ambulatory motor assessment in Parkinson’s disease. *Movement Disorders* 21:34–44, 2006.

106. Ketteringham, L. P., S. A. Neild, R. A. Hyde, R. J. S. Jones, and A. M. D. Smith. Measuring Intention Tremor in Multiple Sclerosis using Inertial Measurement Unit (IMU) Devices. , 2011.

107. Khodakarami, H., P. Farzanehfar, and M. Horne. The Use of Data from the Parkinson’s KinetiGraph to Identify Potential Candidates for Device Assisted Therapies. *Sensors* 19:2241, 2019.

108. Khwaounjoo, P., G. Singh, S. Grenfell, B. Özsoy, M. R. MacAskill, T. J. Anderson, and Y. O. Çakmak. Non-Contact Hand Movement Analysis for Optimal Configuration of Smart Sensors to Capture Parkinson’s Disease Hand Tremor. *Sensors* 22:4613, 2022.

109. Kim, H. B., W. W. Lee, A. Kim, H. J. Lee, H. Y. Park, H. S. Jeon, S. K. Kim, Beomseok. Jeon, and K. S. Park. Wrist sensor-based tremor severity quantification in Parkinson’s disease using convolutional neural network. *Computers in Biology and Medicine* 95:140–146, 2018.

110. Kim, J., T. Wichmann, O. T. Inan, and S. P. DeWeerth. Fitts’ Law Based Performance Metrics to Quantify Tremor in Individuals With Essential Tremor. *IEEE J. Biomed. Health Inform.* 26:2169–2179, 2022.

111. Kim, M. J., E. Naydanova, B. Y. Hwang, K. A. Mills, W. S. Anderson, and Y. Salimpour. Quantification of Parkinson’s Disease Motor Symptoms: A Wireless Motion Sensing Approach. , 2020.doi:10.1109/EMBC44109.2020.9175616

112. Koçer, A., and A. B. Oktay. Nintendo Wii assessment of Hoehn and Yahr score with Parkinson’s disease tremor. *THC* 24:185–191, 2016.

113. Kostikis, N., D. Hristu-Varsakelis, M. Arnaoutoglou, and C. Kotsavasiloglou. A Smartphone-Based Tool for Assessing Parkinsonian Hand Tremor. *IEEE Journal of Biomedical and Health Informatics* 19:1835–1842, 2015.

114. Krishna, R., P. N. Pathirana, M. Horne, L. Power, and D. J. Szmulewicz. Quantitative assessment of cerebellar ataxia, through automated limb functional tests. *Journal of NeuroEngineering and Rehabilitation* 16:31, 2019.

115. Kubben, P. L., M. L. Kuijf, L. P. C. M. Ackermans, A. F. G. Leentjes, and Y. Temel. TREMOR12: An Open-Source Mobile App for Tremor Quantification. *SFN* 94:182–186, 2016.

116. Kulisevsky, J., A. Avila, M. Barbanoj, R. Antonijoan, J. Torres, and R. Arcelus. Levodopa Does Not Aggravate Postural Tremor in Parkinson’s Disease. *Clinical Neuropharmacology* 18:435, 1995.

117. Kuosmanen, E., V. Kan, A. Visuri, J. Vega, Y. Nishiyama, A. K. Dey, S. Harper, and D. Ferreira. Mobile-based Monitoring of Parkinson’s Disease. , 2018.doi:10.1145/3282894.3289737

118. Kuosmanen, E., F. Wolling, J. Vega, V. Kan, Y. Nishiyama, S. Harper, K. V. Laerhoven, S. Hosio, and D. Ferreira. Smartphone-Based Monitoring of Parkinson Disease: Quasi-Experimental Study to Quantify Hand Tremor Severity and Medication Effectiveness. *JMIR mHealth and uHealth* 8:e21543, 2020.

119. Kwon, D.-Y., Y.-R. Kwon, Y.-H. Choi, G.-M. Eom, J. Ko, and J.-W. Kim. Quantitative measures of postural tremor at the upper limb joints in patients with essential tremor. *THC* 28:499–507, 2020.

120. Kwon, D.-Y., Y.-R. Kwon, J. Ko, and J.-W. Kim. Comparison of resting tremor at the upper limb joints between patients with Parkinson’s disease and scans without evidence of dopaminergic deficit. *THC* 31:515–523, 2023.

121. Kwon, Y.-R., G.-M. Eom, J. Ko, and J.-W. Kim. Quantitative analysis of essential tremor during clinical spiral drawing task using gyro sensors. *J. Mech. Med. Biol.* 21:2140050, 2021.

122. Lambrecht, S., J. A. Gallego, E. Rocon, and J. L. Pons. Automatic real-time monitoring and assessment of tremor parameters in the upper limb from orientation data. *Frontiers in Neuroscience* 8:, 2014.

123. Lee, H. J., W. W. Lee, S. K. Kim, H. Park, H. S. Jeon, H. B. Kim, B. S. Jeon, and K. S. Park. Tremor frequency characteristics in Parkinson’s disease under resting-state and stress-state conditions. *Journal of the Neurological Sciences* 362:272–277, 2016.

124. Legrand, A. P., I. Rivals, A. Richard, E. Apartis, E. Roze, M. Vidailhet, S. Meunier, and E. Hainque. New insight in spiral drawing analysis methods – Application to action tremor quantification. *Clinical Neurophysiology* 128:1823–1834, 2017.

125. LeMoyne, R., T. Mastroianni, M. Cozza, C. Coroian, and W. Grundfest. Implementation of an iPhone for characterizing Parkinson’s disease tremor through a wireless accelerometer application. , 2010.doi:10.1109/IEMBS.2010.5627240

126. Li, M. H., T. A. Mestre, S. H. Fox, and B. Taati. Vision-based assessment of parkinsonism and levodopa-induced dyskinesia with pose estimation. *Journal of NeuroEngineering and Rehabilitation* 15:97, 2018.

127. Li, Y., Z. Wang, and H. Dai. Improved Parkinsonian tremor quantification based on automatic label modification and SVM with RBF kernel. *Physiol. Meas.* 44:025003, 2023.

128. Li, Y., J. Yin, S. Liu, B. Xue, C. Shokoohi, G. Ge, M. Hu, T. Li, X. Tao, Z. Rao, F. Meng, H. Shi, X. Ji, P. Servati, X. Xiao, and J. Chen. Learning Hand Kinematics for Parkinson’s Disease Assessment Using a Multimodal Sensor Glove. *Advanced Science* 2206982, 2023.doi:10.1002/advs.202206982

129. Lima, A. L. S. de, T. Hahn, L. J. W. Evers, N. M. de Vries, E. Cohen, M. Afek, L. Bataille, M. Daeschler, K. Claes, B. Boroojerdi, D. Terricabras, M. A. Little, H. Baldus, B. R. Bloem, and M. J. Faber. Feasibility of large-scale deployment of multiple wearable sensors in Parkinson’s disease. *PLOS ONE* 12:e0189161, 2017.

130. de Lima, E. R., A. O. Andrade, J. L. Pons, P. Kyberd, and S. J. Nasuto. Empirical mode decomposition: a novel technique for the study of tremor time series. *Med Bio Eng Comput* 44:569–582, 2006.

131. Lin, F., Z. Wang, H. Zhao, S. Qiu, R. Liu, X. Shi, C. Wang, and W. Yin. Hand Movement Recognition and Salient Tremor Feature Extraction with Wearable Devices in Parkinson’s Patients. *IEEE Transactions on Cognitive and Developmental Systems* 1–1, 2023.doi:10.1109/TCDS.2023.3266812

132. Lipsmeier, F., C. Simillion, A. Bamdadian, R. Tortelli, L. M. Byrne, Y.-P. Zhang, D. Wolf, A. V. Smith, C. Czech, C. Gossens, P. Weydt, S. A. Schobel, F. B. Rodrigues, E. J. Wild, and M. Lindemann. A Remote Digital Monitoring Platform to Assess Cognitive and Motor Symptoms in Huntington Disease: Cross-sectional Validation Study. *Journal of Medical Internet Research* 24:e32997, 2022.

133. Lipsmeier, F., K. I. Taylor, T. Kilchenmann, D. Wolf, A. Scotland, J. Schjodt-Eriksen, W.-Y. Cheng, I. Fernandez-Garcia, J. Siebourg-Polster, L. Jin, J. Soto, L. Verselis, F. Boess, M. Koller, M. Grundman, A. U. Monsch, R. B. Postuma, A. Ghosh, T. Kremer, C. Czech, C. Gossens, and M. Lindemann. Evaluation of smartphone-based testing to generate exploratory outcome measures in a phase 1 Parkinson’s disease clinical trial. *Movement Disorders* 33:1287–1297, 2018.

134. Lipsmeier, F., K. I. Taylor, R. B. Postuma, E. Volkova-Volkmar, T. Kilchenmann, B. Mollenhauer, A. Bamdadian, W. L. Popp, W.-Y. Cheng, Y.-P. Zhang, D. Wolf, J. Schjodt-Eriksen, A. Boulay, H. Svoboda, W. Zago, G. Pagano, and M. Lindemann. Reliability and validity of the Roche PD Mobile Application for remote monitoring of early Parkinson’s disease. *Sci Rep* 12:12081, 2022.

135. Liu, S., H. Yuan, J. Liu, H. Lin, C. Yang, and X. Cai. Comprehensive analysis of resting tremor based on acceleration signals of patients with Parkinson’s disease. *THC* 30:895–907, 2022.

136. Loaiza Duque, J. D., A. M. González-Vargas, A. J. Sánchez Egea, and H. A. González Rojas. Using Machine Learning and Accelerometry Data for Differential Diagnosis of Parkinson’s Disease and Essential Tremor. , 2019.doi:10.1007/978-3-030-31019-6_32

137. Locatelli, P., and D. Alimonti. Differentiating essential tremor and Parkinson’s disease using a wearable sensor — A pilot study. , 2017.doi:10.1109/IWASI.2017.7974254

138. Locatelli, P., D. Alimonti, G. Traversi, and V. Re. Classification of Essential Tremor and Parkinson’s Tremor Based on a Low-Power Wearable Device. *Electronics* 9:1695, 2020.

139. Lonini, L., A. Dai, N. Shawen, T. Simuni, C. Poon, L. Shimanovich, M. Daeschler, R. Ghaffari, J. A. Rogers, and A. Jayaraman. Wearable sensors for Parkinson’s disease: which data are worth collecting for training symptom detection models. *npj Digital Med* 1:1–8, 2018.

140. López-Blanco, R., M. A. Velasco, A. Méndez-Guerrero, J. P. Romero, M. D. del Castillo, J. I. Serrano, J. Benito-León, F. Bermejo-Pareja, and E. Rocon. Essential tremor quantification based on the combined use of a smartphone and a smartwatch: The NetMD study. *Journal of Neuroscience Methods* 303:95–102, 2018.

141. López-Blanco, R., M. A. Velasco, A. Méndez-Guerrero, J. P. Romero, M. D. del Castillo, J. I. Serrano, E. Rocon, and J. Benito-León. Smartwatch for the analysis of rest tremor in patients with Parkinson’s disease. *Journal of the Neurological Sciences* 401:37–42, 2019.

142. Lugo, G., M. Ibarra-Manzano, F. Ba, and I. Cheng. Virtual reality and hand tracking system as a medical tool to evaluate patients with parkinson’s. , 2017.doi:10.1145/3154862.3154924

143. Lunardini, F., D. D. Febbo, M. Malavolti, M. Cid, M. Serra, L. Piccini, A. L. G. Pedrocchi, N. A. Borghese, and S. Ferrante. A Smart Ink Pen for the Ecological Assessment of Age-Related Changes in Writing and Tremor Features. *IEEE Transactions on Instrumentation and Measurement* 70:1–13, 2021.

144. Ma, C., D. Li, L. Pan, X. Li, C. Yin, A. Li, Z. Zhang, and R. Zong. Quantitative assessment of essential tremor based on machine learning methods using wearable device. *Biomedical Signal Processing and Control* 71:103244, 2022.

145. Ma, C., P. Zhang, J. Wang, J. Zhang, L. Pan, X. Li, C. Yin, A. Li, R. Zong, and Z. Zhang. Objective quantification of the severity of postural tremor based on kinematic parameters: A multi-sensory fusion study. *Computer Methods and Programs in Biomedicine* 219:106741, 2022.

146. Mahadevan, N., C. Demanuele, H. Zhang, D. Volfson, B. Ho, M. K. Erb, and S. Patel. Development of digital biomarkers for resting tremor and bradykinesia using a wrist-worn wearable device. *npj Digit. Med.* 3:1–12, 2020.

147. Maldonado-Naranjo, A., M. M. Koop, O. Hogue, J. Alberts, and A. Machado. Kinematic Metrics from a Wireless Stylus Quantify Tremor and Bradykinesia in Parkinson’s Disease. *Parkinson’s Disease* 2019:1–9, 2019.

148. Marino, S., E. Cartella, N. Donato, N. Muscarà, C. Sorbera, V. Cimino, S. De Salvo, K. Micchìa, G. Silvestri, A. Bramanti, and G. Di Lorenzo. Quantitative assessment of Parkinsonian tremor by using biosensor device. *Medicine* 98:e17897, 2019.

149. Matsumoto, J. Y., D. W. Dodick, L. N. Stevens, R. C. Newman, P. E. Caskey, and W. Fjerstad. Three-dimensional measurement of essential tremor. *Mov. Disord.* 14:288–294, 1999.

150. Mcgurrin, P., J. Mcnames, T. Wu, M. Hallett, and D. Haubenberger. Quantifying Tremor in Essential Tremor Using Inertial Sensors—Validation of an Algorithm. *IEEE J. Transl. Eng. Health Med.* 9:1–10, 2021.

151. Mera, T. O., M. A. Burack, and J. P. Giuffrida. Quantitative assessment of levodopa-induced dyskinesia using automated motion sensing technology. , 2012.doi:10.1109/EMBC.2012.6345894

152. Messan, K. S., L. Pham, T. Harris, Y. Kim, V. Morgan, P. Kosa, and B. Bielekova. Assessment of Smartphone-Based Spiral Tracing in Multiple Sclerosis Reveals Intra-Individual Reproducibility as a Major Determinant of the Clinical Utility of the Digital Test. *Frontiers in Medical Technology* 3:, 2022.

153. Milanov, I. Electromyographic differentiation of tremors. *Clinical Neurophysiology* 112:1626–1632, 2001.

154. Mitsui, Y., T. T. Zin, N. Ishii, and H. Mochizuki. Imaging Tremor Quantification for Neurological Disease Diagnosis. *Sensors* 20:6684, 2020.

155. Molparia, B., B. Schrader, E. Cohen, J. Wagner, S. Gupta, S. Gould, N. Hwynn, E. Spencer, and A. Torkamani. Combined accelerometer and genetic analysis to differentiate essential tremor from Parkinson’s disease. *PeerJ* 6:e5308, 2018.

156. Montalban, X., J. Graves, L. Midaglia, P. Mulero, L. Julian, M. Baker, J. Schadrack, C. Gossens, M. Ganzetti, A. Scotland, F. Lipsmeier, J. van Beek, C. Bernasconi, S. Belachew, M. Lindemann, and S. L. Hauser. A smartphone sensor-based digital outcome assessment of multiple sclerosis. *Mult Scler* 28:654–664, 2022.

157. Morgan, M. H., R. L. Hewer, and R. Cooper. Intention tremor--a method of measurement. *Journal of Neurology, Neurosurgery & Psychiatry* 38:253–258, 1975.

158. Mostile, G., J. P. Giuffrida, O. R. Adam, A. Davidson, and J. Jankovic. Correlation between Kinesia system assessments and clinical tremor scores in patients with essential tremor. *Movement Disorders* 25:1938–1943, 2010.

159. Musab, R., A. As’arry, K. A. M. Rezali, N. A. A. Jalil, R. M. K. R. Ahmad, and M. Z. M. Zain. Tremor Quantification and its Measurements Using Shimmer. *J. Phys.: Conf. Ser.* 1262:012024, 2019.

160. Muthuraman, M., G. Deuschl, A. R. Anwar, K. G. Mideksa, F. von Helmolt, and S. A. Schneider. Essential and aging-related tremor: Differences of central control. *Movement Disorders* 30:1673–1680, 2015.

161. Muthuraman, M., U. Heute, K. Arning, A. R. Anwar, R. Elble, G. Deuschl, and J. Raethjen. Oscillating central motor networks in pathological tremors and voluntary movements. What makes the difference? *NeuroImage* 60:1331–1339, 2012.

162. Muthuraman, M., J. Raethjen, N. Koirala, A. R. Anwar, K. G. Mideksa, R. Elble, S. Groppa, and G. Deuschl. Cerebello-cortical network fingerprints differ between essential, Parkinson’s and mimicked tremors. *Brain* 141:1770–1781, 2018.

163. Niazmand, K., K. Tonn, A. Kalaras, U. M. Fietzek, J. H. Mehrkens, and T. C. Lueth. Quantitative evaluation of Parkinson’s disease using sensor based smart glove. , 2011.doi:10.1109/CBMS.2011.5999113

164. Nisticò, R., D. Pirritano, M. Salsone, F. Novellino, F. D. Giudice, M. Morelli, M. Trotta, G. Bilotti, F. Condino, A. Cherubini, P. Valentino, and A. Quattrone. Synchronous pattern distinguishes resting tremor associated with essential tremor from rest tremor of Parkinson’s disease. *Parkinsonism & Related Disorders* 17:30–33, 2011.

165. Nisticò, R., A. Quattrone, M. Crasà, M. De Maria, B. Vescio, and A. Quattrone. Evaluation of rest tremor in different positions in Parkinson’s disease and essential tremor plus. *Neurol Sci* 43:3621–3627, 2022.

166. van den Noort, J. C., R. Verhagen, K. J. van Dijk, P. H. Veltink, M. C. P. M. Vos, R. M. A. de Bie, L. J. Bour, and C. T. Heida. Quantification of Hand Motor Symptoms in Parkinson’s Disease: A Proof-of-Principle Study Using Inertial and Force Sensors. *Ann Biomed Eng* 45:2423–2436, 2017.

167. Norman, K. E., R. Edwards, and A. Beuter. The measurement of tremor using a velocity transducer: comparison to simultaneous recordings using transducers of displacement, acceleration and muscle activity. *Journal of Neuroscience Methods* 92:41–54, 1999.

168. Oliveira, F. H. M., A. G. Rabelo, L. M. D. Luiz, A. A. Pereira, M. F. Vieira, and A. O. Andrade. On the Use of Non-Contact Capacitive Sensors for the Assessment of Postural Hand Tremor of Individuals with Parkinson’s Disease. , 2019.doi:10.1109/EMBC.2019.8856746

169. O’Suilleabhain, P. E., and R. B. Dewey Jr. Validation for tremor quantification of an electromagnetic tracking device. *Movement Disorders* 16:265–271, 2001.

170. Oyama, G., M. Burq, T. Hatano, W. J. Marks, R. Kapur, J. Fernandez, K. Fujikawa, Y. Furusawa, K. Nakatome, E. Rainaldi, C. Chen, K. C. Ho, T. Ogawa, H. Kamo, Y. Oji, H. Takeshige-Amano, D. Taniguchi, R. Nakamura, F. Sasaki, S. Ueno, K. Shiina, A. Hattori, N. Nishikawa, M. Ishiguro, S. Saiki, A. Hayashi, M. Motohashi, and N. Hattori. Analytical and clinical validity of wearable, multi-sensor technology for assessment of motor function in patients with Parkinson’s disease in Japan. *Sci Rep* 13:3600, 2023.

171. Pahwa, R., F. Bergquist, M. Horne, and M. E. Minshall. Objective measurement in Parkinson’s disease: a descriptive analysis of Parkinson’s symptom scores from a large population of patients across the world using the Personal KinetiGraph®. *Journal of Clinical Movement Disorders* 7:5, 2020.

172. Pan, D., R. Dhall, A. Lieberman, and D. B. Petitti. A Mobile Cloud-Based Parkinson’s Disease Assessment System for Home-Based Monitoring. *JMIR mHealth uHealth* 3:e29, 2015.

173. Pan, M.-K., Y.-S. Li, S.-B. Wong, C.-L. Ni, Y.-M. Wang, W.-C. Liu, L.-Y. Lu, J.-C. Lee, E. P. Cortes, J.-P. G. Vonsattel, Q. Sun, E. D. Louis, P. L. Faust, and S.-H. Kuo. Cerebellar oscillations driven by synaptic pruning deficits of cerebellar climbing fibers contribute to tremor pathophysiology. *Science Translational Medicine* 12:eaay1769, 2020.

174. Pang, Y., J. Christenson, F. Jiang, T. Lei, R. Rhoades, D. Kern, J. A. Thompson, and C. Liu. Automatic detection and quantification of hand movements toward development of an objective assessment of tremor and bradykinesia in Parkinson’s disease. *Journal of Neuroscience Methods* 333:108576, 2020.

175. Papapetropoulos, S., J. R. Jagid, C. Sengun, C. Singer, and B. V. Gallo. Objective monitoring of tremor and bradykinesia during DBS surgery for Parkinson disease. *Neurology* 70:1244–1249, 2008.

176. Papapetropoulos, S., H. L. Katzen, B. K. Scanlon, A. Guevara, C. Singer, and B. E. Levin. Objective Quantification of Neuromotor Symptoms in Parkinson’s Disease: Implementation of a Portable, Computerized Measurement Tool. *Parkinson’s Disease* 2010:1–6, 2010.

177. Park, K. W., H. J. Wu, T. Yu, R. Mahal, M. S. Mirian, and M. J. McKeown. Potential Pitfalls of Remote and Automated Video Assessments of Movements Disorders. *Movement Disorders* 38:504–506, 2023.

178. Patel, S., K. Lorincz, R. Hughes, N. Huggins, J. Growdon, D. Standaert, M. Akay, J. Dy, M. Welsh, and P. Bonato. Monitoring Motor Fluctuations in Patients With Parkinson’s Disease Using Wearable Sensors. *IEEE Transactions on Information Technology in Biomedicine* 13:864–873, 2009.

179. Patel, V., M. Burns, M. Pourfar, A. Mogilner, D. Kondziolka, and R. Vinjamuri. QAPD: An integrated system to quantify symptoms of Parkinson’s disease. , 2016.doi:10.1109/EMBC.2016.7591073

180. Pedrosa, D. J., C. Nelles, P. Brown, L. J. Volz, E. A. Pelzer, M. Tittgemeyer, J.-S. Brittain, and L. Timmermann. The differentiated networks related to essential tremor onset and its amplitude modulation after alcohol intake. *Experimental Neurology* 297:50–61, 2017.

181. Perera, T., W.-L. Lee, S. A. C. Yohanandan, A.-L. Nguyen, B. Cruse, F. M. C. Boonstra, G. Noffs, A. P. Vogel, S. C. Kolbe, H. Butzkueven, A. Evans, and A. Van Der Walt. Validation of a precision tremor measurement system for multiple sclerosis. *Journal of Neuroscience Methods* 311:377–384, 2019.

182. Peres, L. B., B. C. Calil, A. P. S. P. B. Da Silva, V. C. Dionísio, M. F. Vieira, A. De Oliveira Andrade, and A. A. Pereira. Discrimination between healthy and patients with Parkinson’s disease from hand resting activity using inertial measurement unit. *BioMed Eng OnLine* 20:50, 2021.

183. Piboolnurak, P., N. Rothey, A. Ahmed, B. Ford, Q. Yu, D. Xu, and S. L. Pullman. Psychogenic tremor disorders identified using tree-based statistical algorithms and quantitative tremor analysis. *Movement Disorders* 20:1543–1549, 2005.

184. Powers, R., M. Etezadi-Amoli, E. M. Arnold, S. Kianian, I. Mance, M. Gibiansky, D. Trietsch, A. S. Alvarado, J. D. Kretlow, T. M. Herrington, S. Brillman, N. Huang, P. T. Lin, H. A. Pham, and A. V. Ullal. Smartwatch inertial sensors continuously monitor real-world motor fluctuations in Parkinson’s disease. *Science Translational Medicine* 13:eabd7865, 2021.

185. Pradhan, S. D., B. R. Brewer, G. E. Carvell, P. J. Sparto, A. Delitto, and Y. Matsuoka. Assessment of Fine Motor Control in Individuals with Parkinson’s Disease Using Force Tracking with a Secondary Cognitive Task. *Journal of Neurologic Physical Therapy* 34:32–40, 2010.

186. Pulliam, C. L., S. R. Eichenseer, C. G. Goetz, O. Waln, C. B. Hunter, J. Jankovic, D. E. Vaillancourt, J. P. Giuffrida, and D. A. Heldman. Continuous in-home monitoring of essential tremor. *Parkinsonism & Related Disorders* 20:37–40, 2014.

187. Pulliam, C. L., D. A. Heldman, E. B. Brokaw, T. O. Mera, Z. K. Mari, and M. A. Burack. Continuous Assessment of Levodopa Response in Parkinson’s Disease Using Wearable Motion Sensors. *IEEE Transactions on Biomedical Engineering* 65:159–164, 2018.

188. Pullman, S. L. Spiral Analysis: A New Technique for Measuring Tremor With a Digitizing Tablet. *Movement Disorders* 13:85–89, 1998.

189. Rabelo, A., J. P. Folador, A. P. Bittar, L. Maire, S. Costa, A. Rueda, S. Krishnan, V. Lima, R. M. A. Almeida, and A. O. Andrade. Low Amplitude Hand Rest Tremor Assessment in Parkinson’s Disease Based on Linear and Nonlinear Methods. , 2022.doi:10.1007/978-3-030-70601-2_46

190. Rahimi, F., C. Bee, D. Debicki, A. C. Roberts, P. Bapat, and M. Jog. Effectiveness of BoNT A in Parkinson’s Disease Upper Limb Tremor Management. *Can. J. Neurol. Sci.* 40:663–669, 2013.

191. Ricci, M., G. D. Lazzaro, V. Errico, A. Pisani, F. Giannini, and G. Saggio. The Impact of Wearable Electronics in Assessing the Effectiveness of Levodopa Treatment in Parkinson’s Disease. *IEEE J. Biomed. Health Inform.* 26:2920–2928, 2022.

192. Rigas, G., D. Gatsios, D. I. Fotiadis, M. Chondrogiorgi, C. Tsironis, S. Konitsiotis, G. Gentile, A. Marcante, and A. Antonini. Tremor UPDRS estimation in home environment. , 2016.doi:10.1109/EMBC.2016.7591517

193. Rigas, G., A. T. Tzallas, M. G. Tsipouras, P. Bougia, E. E. Tripoliti, D. Baga, D. I. Fotiadis, S. G. Tsouli, and S. Konitsiotis. Assessment of Tremor Activity in the Parkinson’s Disease Using a Set of Wearable Sensors. *IEEE Transactions on Information Technology in Biomedicine* 16:478–487, 2012.

194. Riviere, C. N., S. G. Reich, and N. V. Thakor. Adaptive Fourier modeling for quantification of tremor. *Journal of Neuroscience Methods* 74:77–87, 1997.

195. Roy, S. H., B. T. Cole, L. D. Gilmore, C. J. De Luca, C. A. Thomas, M. M. Saint-Hilaire, and S. H. Nawab. High-resolution tracking of motor disorders in Parkinson’s disease during unconstrained activity. *Movement Disorders* 28:1080–1087, 2013.

196. Rozman, J., A. Bartolić, and S. Ribarič. A new method for selective measurement of joint movement in hand tremor in Parkinson’s disease patients. *Journal of Medical Engineering & Technology* 31:305–311, 2007.

197. Sahin, G., P. Halje, S. Uzun, A. Jakobsson, and P. Petersson. Tremor evaluation using smartphone accelerometry in standardized settings. *Front. Neurosci.* 16:861668, 2022.

198. Salarian, A., H. Russmann, C. Wider, P. R. Burkhard, F. J. G. Vingerhoets, and K. Aminian. Quantification of Tremor and Bradykinesia in Parkinson’s Disease Using a Novel Ambulatory Monitoring System. *IEEE Transactions on Biomedical Engineering* 54:313–322, 2007.

199. Sanchez-Perez, L. A., L. P. Sanchez-Fernandez, A. Shaout, J. M. Martinez-Hernandez, and M. J. Alvarez-Noriega. Rest tremor quantification based on fuzzy inference systems and wearable sensors. *International Journal of Medical Informatics* 114:6–17, 2018.

200. Santiago, A., J. W. Langston, R. Gandhy, R. Dhall, S. Brillman, L. Rees, and C. Barlow. Qualitative Evaluation of the Personal KinetiGraphTM Movement Recording System in a Parkinson’s Clinic. *J Parkinsons Dis* 9:207–219.

201. Saraguro, W., B. Barzallo, J. Guillermo, A. García-Cedeño, A. Soto, D. Rivas, R. Clotet, and M. Huerta. Analysis of hand movements in patients with Parkinson’s Disease using Kinect. , 2019.doi:10.1109/HealthCom46333.2019.9009589

202. Scanlon, B. K., B. E. Levin, D. A. Nation, H. L. Katzen, A. Guevara-Salcedo, C. Singer, and S. Papapetropoulos. An accelerometry-based study of lower and upper limb tremor in Parkinson’s disease. *Journal of Clinical Neuroscience* 20:827–830, 2013.

203. Senova, S., D. Querlioz, C. Thiriez, P. Jedynak, B. Jarraya, and S. Palfi. Using the Accelerometers Integrated in Smartphones to Evaluate Essential Tremor. *Stereotactic and functional neurosurgery* 93:94–101, 2015.

204. Shaikh, A. G., H. A. Jinnah, R. M. Tripp, L. M. Optican, S. Ramat, F. A. Lenz, and D. S. Zee. Irregularity distinguishes limb tremor in cervical dystonia from essential tremor. *Journal of Neurology, Neurosurgery & Psychiatry* 79:187–189, 2008.

205. Shawen, N., M. K. O’Brien, S. Venkatesan, L. Lonini, T. Simuni, J. L. Hamilton, R. Ghaffari, J. A. Rogers, and A. Jayaraman. Role of data measurement characteristics in the accurate detection of Parkinson’s disease symptoms using wearable sensors. *Journal of NeuroEngineering and Rehabilitation* 17:52, 2020.

206. Sigcha, L., I. Pavón, N. Costa, S. Costa, M. Gago, P. Arezes, J. M. López, and G. De Arcas. Automatic Resting Tremor Assessment in Parkinson’s Disease Using Smartwatches and Multitask Convolutional Neural Networks. *Sensors* 21:291, 2021.

207. Sisti, J. A., B. Christophe, A. R. Seville, A. L. A. Garton, V. P. Gupta, A. J. Bandin, Q. Yu, and S. L. Pullman. Computerized spiral analysis using the iPad. *Journal of Neuroscience Methods* 275:50–54, 2017.

208. Smeja, M., F. Foerster, G. Fuchs, D. Emmans, A. Hornig, and J. Fahrenberg. 24-h Assessment of Tremor Activity and Posture in Parkinson’s Disease by Multi-Channel Accelerometry. *Journal of Psychophysiology* , 2006.at <https://econtent.hogrefe.com/doi/10.1027//0269-8803.13.4.245>

209. Smid, A., J. W. J. Elting, J. M. C. Van Dijk, B. Otten, D. L. M. Oterdoom, K. Tamasi, T. Heida, T. Van Laar, and G. Drost. Intraoperative Quantification of MDS-UPDRS Tremor Measurements Using 3D Accelerometry: A Pilot Study. *JCM* 11:2275, 2022.

210. Song, P., S. Li, S. Wang, H. Wei, H. Lin, and Y. Wang. Repetitive transcranial magnetic stimulation of the cerebellum improves ataxia and cerebello-fronto plasticity in multiple system atrophy: a randomized, double-blind, sham-controlled and TMS-EEG study. *aging* 12:20611–20622, 2020.

211. Spieker, S., A. Boose, S. Breit, and J. Dichgans. Long-Term Measurement of Tremor. *Movement Disorders* 13:81–84, 1998.

212. Spieker, S., V. Ströle, A. Sailer, A. Boose, and J. Dichgans. Validity of long-term electromyography in the quantification of tremor. *Movement Disorders* 12:985–991, 1997.

213. Stanley, K., J. Hagenah, N. Brüggemann, K. Reetz, L. Severt, C. Klein, Q. Yu, C. Derby, S. Pullman, and R. Saunders-Pullman. Digitized spiral analysis is a promising early motor marker for Parkinson Disease. *Parkinsonism & Related Disorders* 16:233–234, 2010.

214. Sun, M., A. Watson, G. Blackwell, W. Jung, S. Wang, K. Koltermann, N. Helm, G. Zhou, L. Cloud, and I. Pretzer-Aboff. TremorSense: Tremor Detection for Parkinson’s Disease Using Convolutional Neural Network. , 2021.doi:10.1109/CHASE52844.2021.00009

215. Surangsrirat, D., C. Thanawattano, R. Pongthornseri, S. Dumnin, C. Anan, and R. Bhidayasiri. Support vector machine classification of Parkinson’s disease and essential tremor subjects based on temporal fluctuation. , 2016.doi:10.1109/EMBC.2016.7592190

216. Synnott, J., Liming Chen, C. D. Nugent, and G. Moore. WiiPD—Objective Home Assessment of Parkinson’s Disease Using the Nintendo Wii Remote. *IEEE Trans. Inform. Technol. Biomed.* 16:1304–1312, 2012.

217. Szumilas, M., K. Lewenstein, E. Ślubowska, S. Szlufik, and D. Koziorowski. A Multimodal Approach to the Quantification of Kinetic Tremor in Parkinson’s Disease. *Sensors* 20:184, 2019.

218. Teskey, W. J. E., M. Elhabiby, and N. El-Sheimy. Inertial Sensing to Determine Movement Disorder Motion Present before and after Treatment. *Sensors* 12:3512–3527, 2012.

219. Teufl, S., J. Preston, F. van Wijck, and B. Stansfield. Quantifying upper limb tremor in people with multiple sclerosis using Fast Fourier Transform based analysis of wrist accelerometer signals. *Journal of Rehabilitation and Assistive Technologies Engineering* 8:2055668320966955, 2021.

220. Thanawattano, C., R. Pongthornseri, C. Anan, S. Dumnin, and R. Bhidayasiri. Temporal fluctuations of tremor signals from inertial sensor: a preliminary study in differentiating Parkinson’s disease from essential tremor. *BioMed Eng OnLine* 14:101, 2015.

221. Thielgen, T., F. Foerster, G. Fuchs, A. Hornig, and J. Fahrenberg. Tremor in Parkinson’s disease: 24-hr monitoring with calibrated accelerometry. *Electromyography and clinical neurophysiology* 44:137–46, 2004.

222. Timmer, J., M. Lauk, and G. Deuschl. Quantitative analysis of tremor time series. *Electroencephalography and Clinical Neurophysiology/Electromyography and Motor Control* 101:461–468, 1996.

223. Toffoli, S., F. Lunardini, M. Parati, M. Gallotta, B. De Maria, L. Longoni, M. E. Dell’Anna, and S. Ferrante. Spiral drawing analysis with a smart ink pen to identify Parkinson’s disease fine motor deficits. *Front. Neurol.* 14:1093690, 2023.

224. Tran, H., K. D. Nguyen, P. N. Pathirana, M. Horne, L. Power, and D. J. Szmulewicz. Multimodal Data Acquisition for the Assessment of Cerebellar Ataxia via Ballistic Tracking. , 2020.doi:10.1109/EMBC44109.2020.9176379

225. Tran, H., P. N. Pathirana, M. Horne, L. Power, and D. J. Szmulewicz. Automated Evaluation of Upper Limb Motor Impairment of Patient with Cerebellar Ataxia. , 2019.doi:10.1109/EMBC.2019.8856330

226. Tsiouris, K. M., D. Gatsios, G. Rigas, D. Miljkovic, B. Koroušić Seljak, M. Bohanec, M. T. Arredondo, A. Antonini, S. Konitsiotis, D. D. Koutsouris, and D. I. Fotiadis. PD_Manager: an mHealth platform for Parkinson’s disease patient management. *Healthcare Technology Letters* 4:102–108, 2017.

227. Tzallas, A. T., M. G. Tsipouras, G. Rigas, D. G. Tsalikakis, E. C. Karvounis, M. Chondrogiorgi, F. Psomadellis, J. Cancela, M. Pastorino, M. T. A. Waldmeyer, S. Konitsiotis, and D. I. Fotiadis. PERFORM: A System for Monitoring, Assessment and Management of Patients with Parkinson’s Disease. *Sensors* 14:21329–21357, 2014.

228. Van Someren, E. J. W., W. A. Van Gool, B. F. M. Vonk, M. Mirmiran, J. D. Speelman, D. A. Bosch, and D. F. Swaab. Ambulatory monitoring of tremor and other movements before and after thalamotomy: A new quantitative technique. *Journal of the Neurological Sciences* 117:16–23, 1993.

229. Van Someren, E. J. W., B. F. M. Vonk, W. A. Thijssen, J. D. Speelman, P. R. Schuurman, M. Mirmiran, and D. F. Swaab. A new actigraph for long-term registration of the duration and intensity of tremor and movement. *IEEE Transactions on Biomedical Engineering* 45:386–395, 1998.

230. Varghese, J., C. M. van Alen, M. Fujarski, G. S. Schlake, J. Sucker, T. Warnecke, and C. Thomas. Sensor Validation and Diagnostic Potential of Smartwatches in Movement Disorders. *Sensors* 21:3139, 2021.

231. Verleger, R., E. Wascher, B. Wauschkuhn, P. Jas´kowski, B. Allouni, P. Trillenberg, and K. Wessel. Consequences of altered cerebellar input for the cortical regulation of motor coordination, as reflected in EEG potentials. *Exp Brain Res* 127:409–422, 1999.

232. Vescio, B., R. Nisticò, A. Augimeri, A. Quattrone, M. Crasà, and A. Quattrone. Development and Validation of a New Wearable Mobile Device for the Automated Detection of Resting Tremor in Parkinson’s Disease and Essential Tremor. *Diagnostics* 11:200, 2021.

233. Wang, S.-Y., T. Z. Aziz, J. F. Stein, and X. Liu. Time–frequency analysis of transient neuromuscular events: dynamic changes in activity of the subthalamic nucleus and forearm muscles related to the intermittent resting tremor. *Journal of Neuroscience Methods* 145:151–158, 2005.

234. Wang, Y., J. Yang, M. Cai, X. Liu, K. Lu, Y. Lou, and Z. Li. Application of optimized convolutional neural networks for early aided diagnosis of essential tremor: Automatic handwriting recognition and feature analysis. *Medical Engineering & Physics* 113:103962, 2023.

235. Western, D. G., S. A. Neild, R. Jones, and A. Davies-Smith. Personalised profiling to identify clinically relevant changes in tremor due to multiple sclerosis. *BMC Medical Informatics and Decision Making* 19:162, 2019.

236. Wile, D. J., R. Ranawaya, and Z. H. T. Kiss. Smart watch accelerometry for analysis and diagnosis of tremor. *Journal of Neuroscience Methods* 230:1–4, 2014.

237. Wilkins, K. B., M. N. Petrucci, Y. Kehnemouyi, A. Velisar, K. Han, G. Orthlieb, M. H. Trager, J. J. O’Day, S. Aditham, and H. Bronte-Stewart. Quantitative Digitography Measures Motor Symptoms and Disease Progression in Parkinson’s Disease. *JPD* 12:1979–1990, 2022.

238. Williams, S., H. Fang, S. D. Relton, D. C. Wong, T. Alam, and J. E. Alty. Accuracy of Smartphone Video for Contactless Measurement of Hand Tremor Frequency. *Movement Disorders Clinical Practice* 8:69–75, 2021.

239. Wong, S.-B., Y.-M. Wang, C.-C. Lin, S. K. Geng, N. Vanegas-Arroyave, S. L. Pullman, S.-H. Kuo, and M.-K. Pan. Cerebellar Oscillations in Familial and Sporadic Essential Tremor. *Cerebellum* 21:425–431, 2022.

240. Woods, A. M., M. Nowostawski, E. A. Franz, and M. Purvis. Parkinson’s disease and essential tremor classification on mobile device. *Pervasive and Mobile Computing* 13:1–12, 2014.

241. Yu Su, C. R. Allen, D. Geng, D. Burn, U. Brechany, G. D. Bell, and R. Rowland. 3-D motion system (“data-gloves”): application for Parkinson’s disease. *IEEE Trans. Instrum. Meas.* 52:662–674, 2003.

242. Yuan, H., S. Liu, J. Liu, H. Lin, C. Yang, X. Cai, L. Zeng, and S. Li. Detection and Quantification of Resting Tremor in Parkinson’s Disease Using Long-Term Acceleration Data. *Mathematical Problems in Engineering* 2021:e5669932, 2021.

243. Zajki-Zechmeister, T. Quantification of tremor severity with a mobile tremor pen. 8, 2020.

244. Zeuner, K. E., R. O. Shoge, S. R. Goldstein, J. M. Dambrosia, and M. Hallett. Accelerometry to distinguish psychogenic from essential or parkinsonian tremor. *Neurology* 61:548–550, 2003.

245. Zham, P., S. P. Arjunan, S. Raghav, and D. K. Kumar. Efficacy of Guided Spiral Drawing in the Classification of Parkinson’s Disease. *IEEE Journal of Biomedical and Health Informatics* 22:1648–1652, 2018.

246. Zhan, A., S. Mohan, C. Tarolli, R. B. Schneider, J. L. Adams, S. Sharma, M. J. Elson, K. L. Spear, A. M. Glidden, M. A. Little, A. Terzis, E. R. Dorsey, and S. Saria. Using Smartphones and Machine Learning to Quantify Parkinson Disease Severity: The Mobile Parkinson Disease Score. *JAMA Neurology* 75:876–880, 2018.

247. Zhang, A., R. San-Segundo, S. Panev, G. Tabor, K. Stebbins, A. Whitford, F. De la Torre, and J. Hodgins. Automated Tremor Detection in Parkinson’s Disease Using Accelerometer Signals. , 2018.doi:10.1145/3278576.3278582

248. Zhang, B., F. Huang, J. Liu, and D. Zhang. A Novel Posture for Better Differentiation Between Parkinson’s Tremor and Essential Tremor. *Frontiers in Neuroscience* 12:, 2018.

249. Zheng, X., A. Vieira, S. L. Marcos, Y. Aladro, and J. Ordieres-Meré. Activity-aware essential tremor evaluation using deep learning method based on acceleration data. *Parkinsonism & Related Disorders* 58:17–22, 2019.

250. Zheng, X., A. Vieira Campos, J. Ordieres-Meré, J. Balseiro, S. Labrador Marcos, and Y. Aladro. Continuous Monitoring of Essential Tremor Using a Portable System Based on Smartwatch. *Frontiers in Neurology* 8:, 2017.

251. Zhou, Y., M. E. Jenkins, M. D. Naish, and A. L. Trejos. Development of a Wearable Tremor Suppression Glove. , 2018.doi:10.1109/BIOROB.2018.8487197

252. Zwartjes, D. G. M., T. Heida, J. P. P. van Vugt, J. A. G. Geelen, and P. H. Veltink. Ambulatory Monitoring of Activities and Motor Symptoms in Parkinson’s Disease. *IEEE Transactions on Biomedical Engineering* 57:2778–2786, 2010.
