# Supplementary material for: Upper limb intention tremor assessment: opportunities and challenges in wearable technology
Source: J Neuroeng Rehabil. 2024 Jan 13;21:8. doi: 10.1186/s12984-023-01302-9 (PMC10787996; doi:10.1186/s12984-023-01302-9)
Supplement: Supplementary file 3 — Additional file 3. Results of literature search and data extraction. This flow diagram shows the number of sources of evidence screened and assessed for eligibility and the number of studies excluded at each stage of the data extraction process. [file 12984_2023_1302_MOESM3_ESM.docx]

**Additional File 3.** Results of literature search and data extraction

Studies removed *before screening*:

Duplicate records removed PUBMED (n = 3)

Studies identified from:

PUBMED (n =650)

SCOPUS (n=1688)

**IDENTIFICATION**

Studies screened

PUBMED (n =647)

SCOPUS (n=1688)

Studies excluded:

PUBMED Reviews or Book Chapters (n = 20)

SCOPUS Reviews or Book Chapters (n = 171)

Studies sought for retrieval

PUBMED (n =627)

SCOPUS (n=1517)

Studies not retrieved:

PUBMED other language than English

(n = 18)

PUBMED other language than English

(n = 143)

**SCREENING**

Studies assessed for eligibility

PUBMED (n = 609)

SCOPUS (n = 1374)

Studies excluded by exclusion criteria (See section 3. Literature search and data extraction)

PUBMED (n =479)

SCOPUS (n = 1322)

Studies in PUBMED and SCOPUS (n = 18)

**INCLUDED**

Studies included in review

(n = 243)

Studies included from screening citations

(n = 79)

*Modified From:*  Page MJ, McKenzie JE, Bossuyt PM, Boutron I, Hoffmann TC, Mulrow CD, et al. The PRISMA 2020 statement: an updated guideline for reporting systematic reviews. BMJ 2021;372:n71. doi: 10.1136/bmj.n71
